# Supplementary material for: CasKAS: direct profiling of genome-wide dCas9 and Cas9 specificity using ssDNA mapping
Source: Genome Biol. 2023 Apr 21;24:85. doi: 10.1186/s13059-023-02930-z (PMC10120127; doi:10.1186/s13059-023-02930-z)
Supplement: Supplementary file 1 — Additional file 1: Supplementary Figure 1. In vitro dCas9 and Cas9 CasKAS profiles around the mouse Nanog locus using the “Nanog-sg2” and “Nanog-sg3” sgRNAs. Supplementary Figure 2. CasKAS signal in vitro is specific to the activity of the dCas9/Cas9 protein combined with its sgRNA. Supplementary Figure 3. CasKAS signal in vitro around the VEGFA gene with the VEGFA sgRNA. Supplementary Figure 4. CasKAS signal in vivo around the VEGFA gene with the VEGFA sgRNA. Supplementary Figure 5. Time course of in vivo CasKAS signal around the EMX1 gene with the EMX1 sgRNA using dCas9. Supplementary Figure 6. Time course of in vivo CasKAS signal around the VEGFA gene with the VEGFA sgRNA using dCas9. Supplementary Figure 7. Time course of in vivo CasKAS signal around the EMX1 gene with the EMX1 sgRNA using active Cas9. Supplementary Figure 8. Time course of in vivo CasKAS signal around the VEGFA gene with the VEGFA sgRNA using active Cas9. Supplementary Figure 9. CasKAS signal in vitro around the CD2 gene with two different sgRNA targeting the gene. Supplementary Figure 10. CasKAS signal in vivo (HEK293 cells, harvested at 48 hours) around the CD2 gene with two different sgRNA targeting the gene. Supplementary Figure 11. CasKAS signal in vitro around the CD90/THY1 gene with two different sgRNA targeting the gene. Supplementary Figure 12. CasKAS signal in vivo (HEK293 cells, harvested at 48 hours) around the CD90/THY1 gene with two different sgRNA targeting the gene. Supplementary Figure 13. CasKAS signal in vitro around the CD45/PTPRC gene with two different sgRNA targeting the gene. Supplementary Figure 14. CasKAS signal in vivo (HEK293 cells, harvested at 48 hours) around the CD45/PTPRC gene with two different sgRNA targeting the gene. Supplementary Figure 15. CasKAS signal in vitro around the CD298/ATP1B3 gene with two different sgRNA targeting the gene. Supplementary Figure 16. CasKAS signal in vivo (HEK293 cells, harvested at 48 hours) around the CD298/ATP1B3 gene with two [file 13059_2023_2930_MOESM1_ESM.pdf]

Supplementary Materials

Supplementary Figures

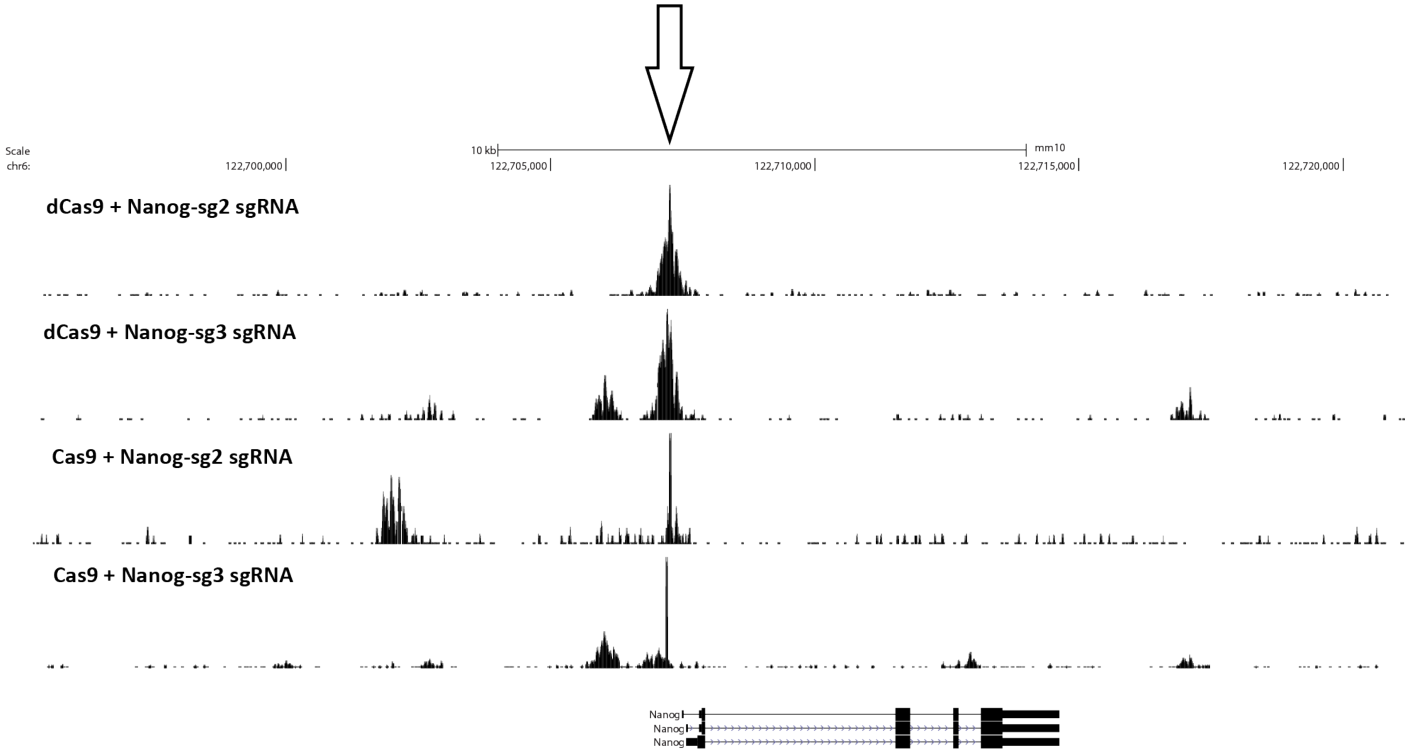

Supplementary Figure 1: ***In vitro*** **dCas9 and Cas9 CasKAS profiles around the mouse** ***Nanog*** **locus using the "Nanog-sg2" and "Nanog-sg3" sgRNAs**.

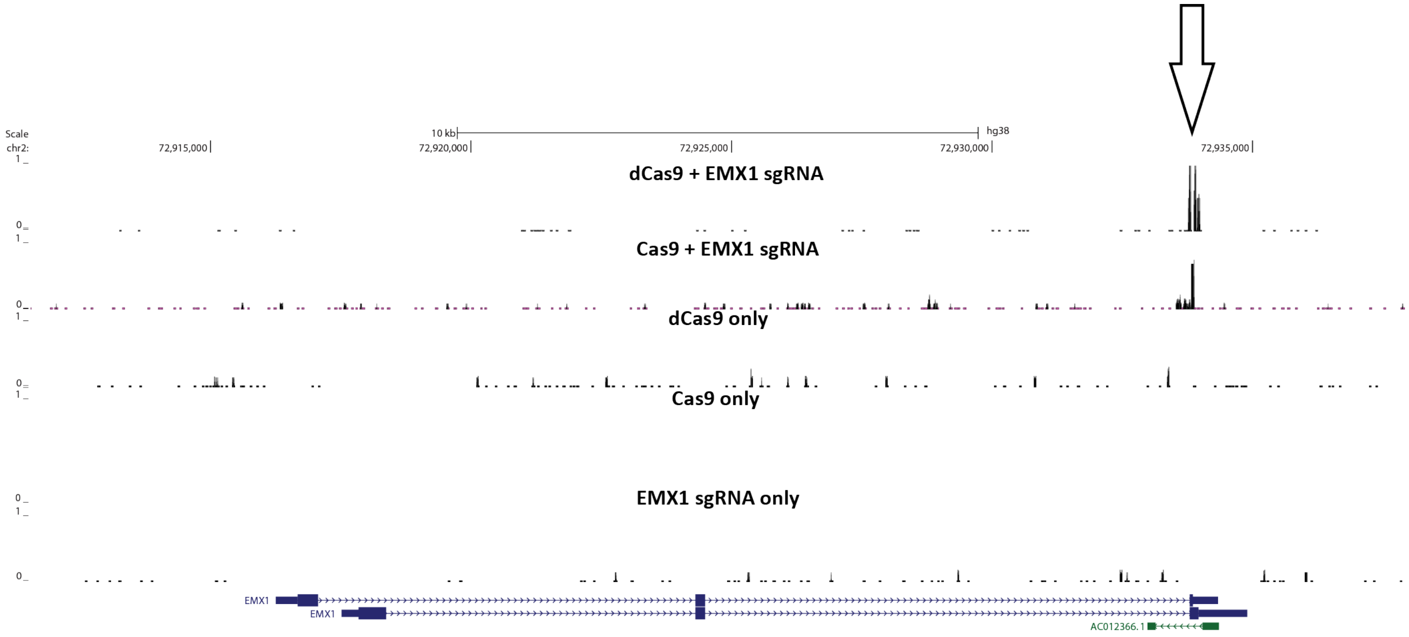

Supplementary Figure 2: CasKAS signal *in vitro* is specific to the activity of the dCas9/Cas9 protein combined with its sgRNA. CasKAS was carried out with the EMX1 sgRNA and with the following combinations of protein and sgRNA: dCas9 + sgRNA, Cas9 + sgRNA, dCas9 alone, Cas9 alone, or sgRNA alone.

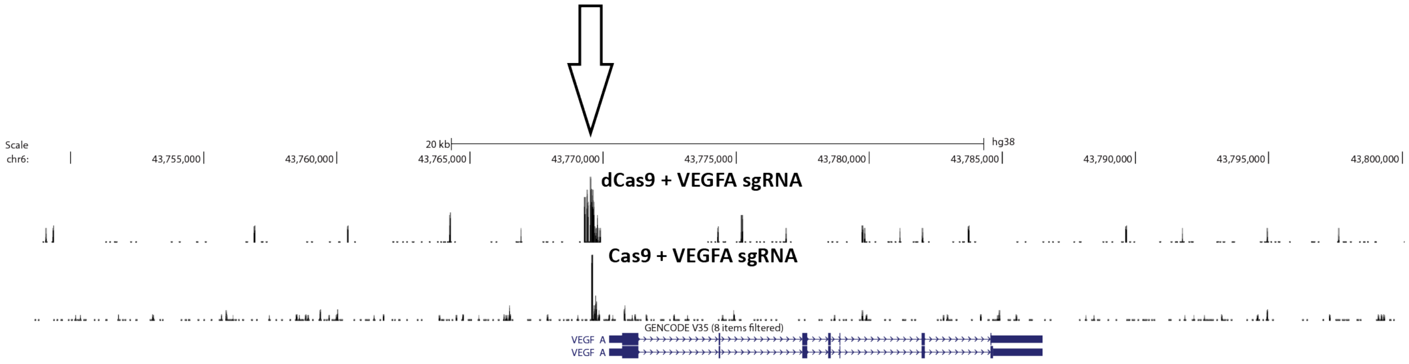

Supplementary Figure 3: CasKAS signal *in vitro* around the *VEGFA* gene with the VEGFA sgRNA.

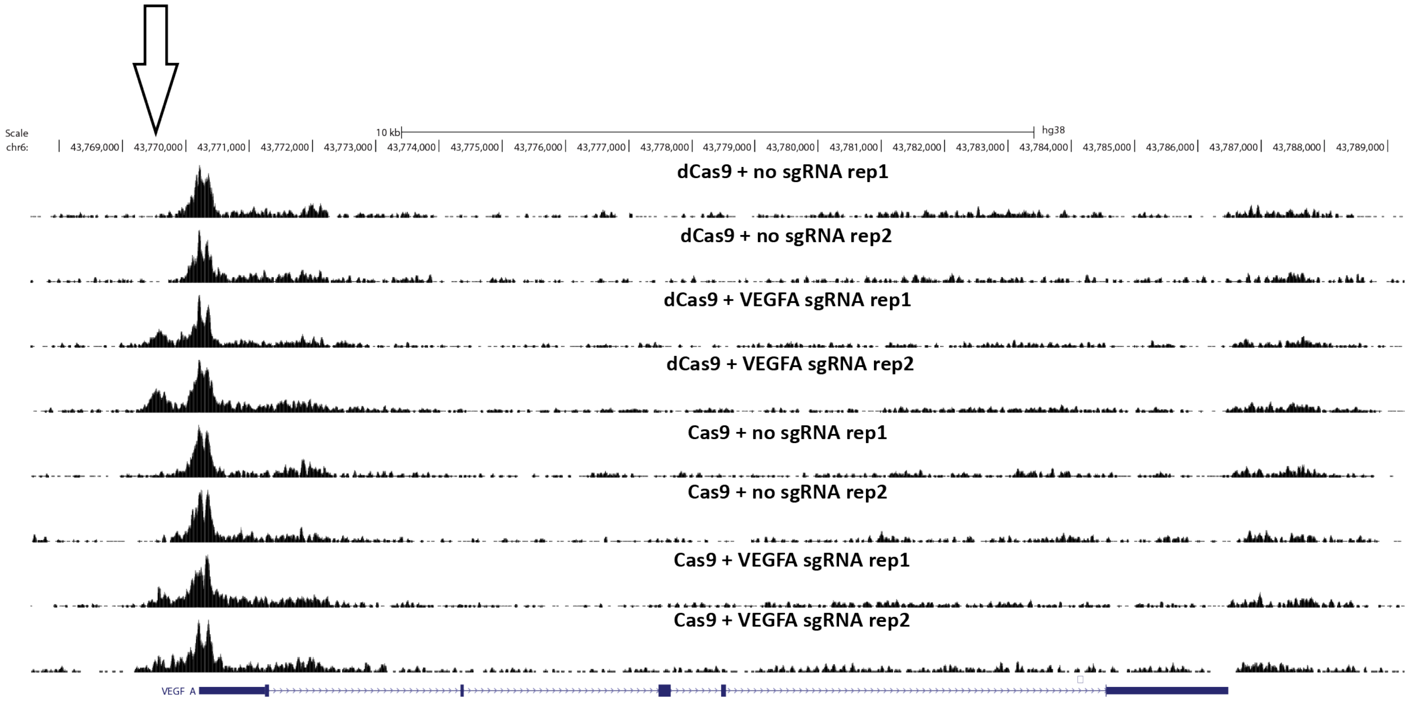

Supplementary Figure 4: CasKAS signal *in vivo* around the *VEGFA* gene with the VEGFA sgRNA.

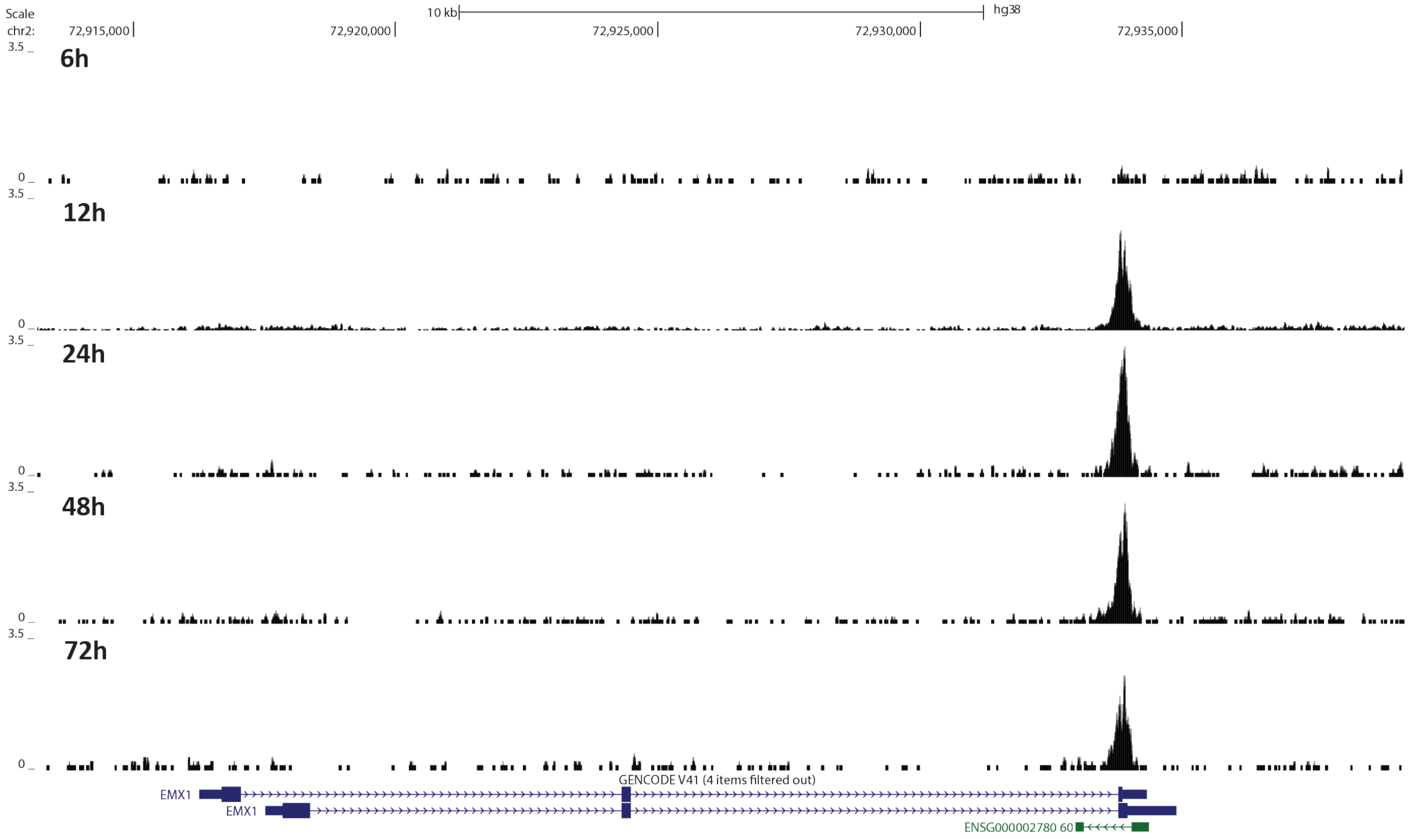

Supplementary Figure 5: Time course of *in vivo* CasKAS signal around the *EMX1* gene with the EMX1 sgRNA using dCas9. HEK293 cells were harvested and KAS-seq carried out at the indicated time points after the initiation of the *in vivo* CasKAS experiment.

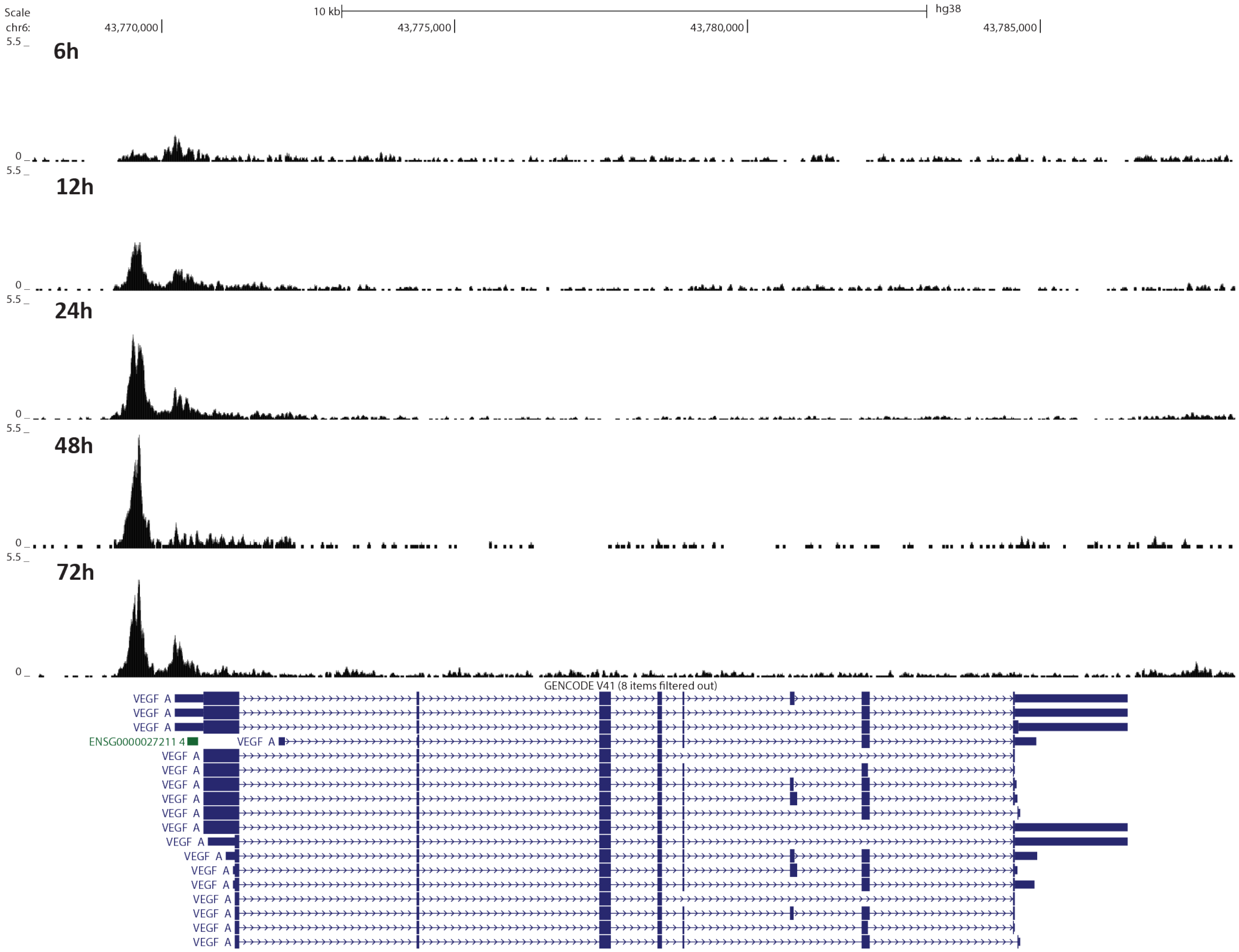

Supplementary Figure 6: Time course of *in vivo* CasKAS signal around the *VEGFA* gene with the VEGFA sgRNA using dCas9. HEK293 cells were harvested and KAS-seq carried out at the indicated time points after the initiation of the *in vivo* CasKAS experiment.

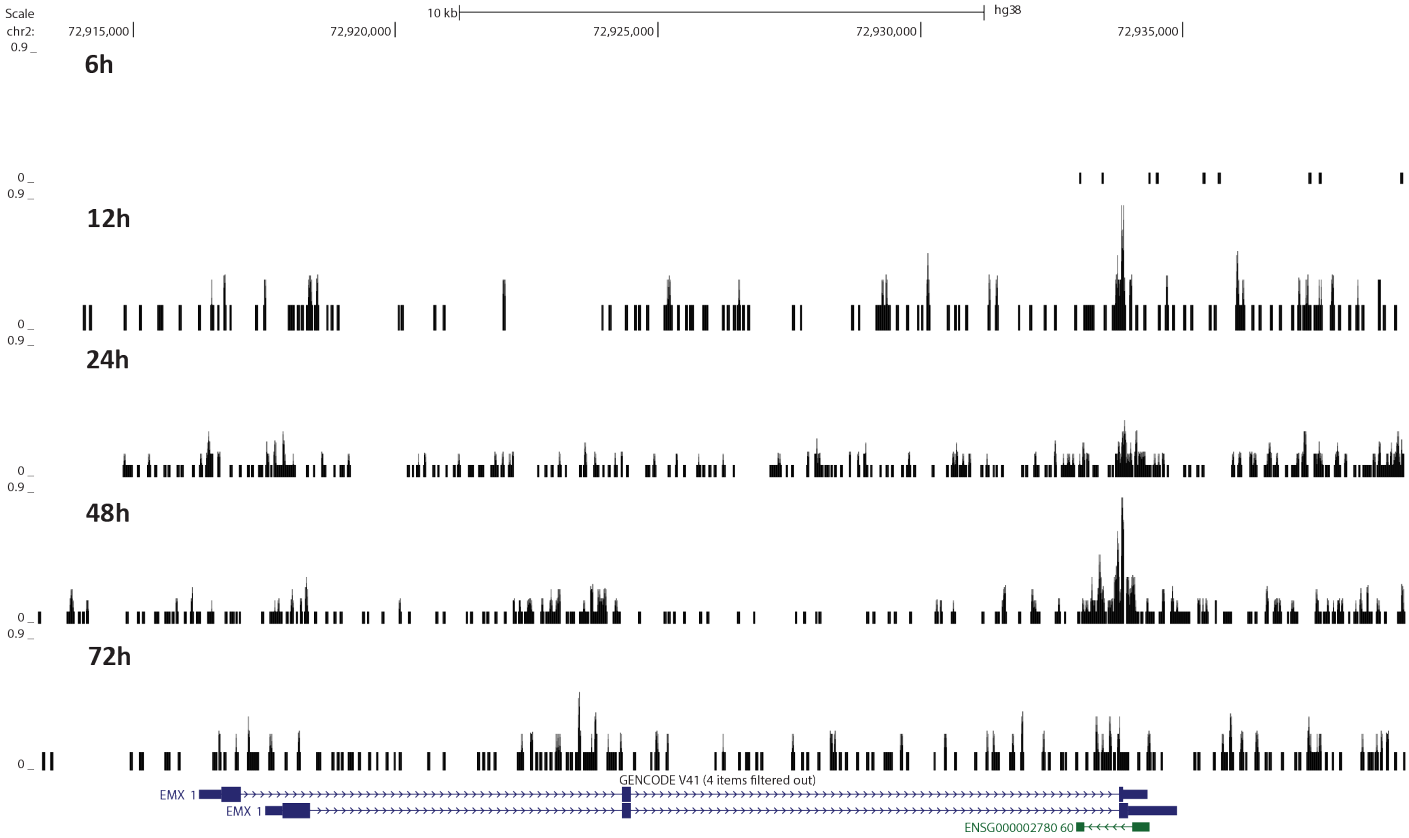

Supplementary Figure 7: Time course of *in vivo* CasKAS signal around the *EMX1* gene with the EMX1 sgRNA using active Cas9. HEK293 cells were harvested and KAS-seq carried out at the indicated time points after the initiation of the *in vivo* CasKAS experiment.

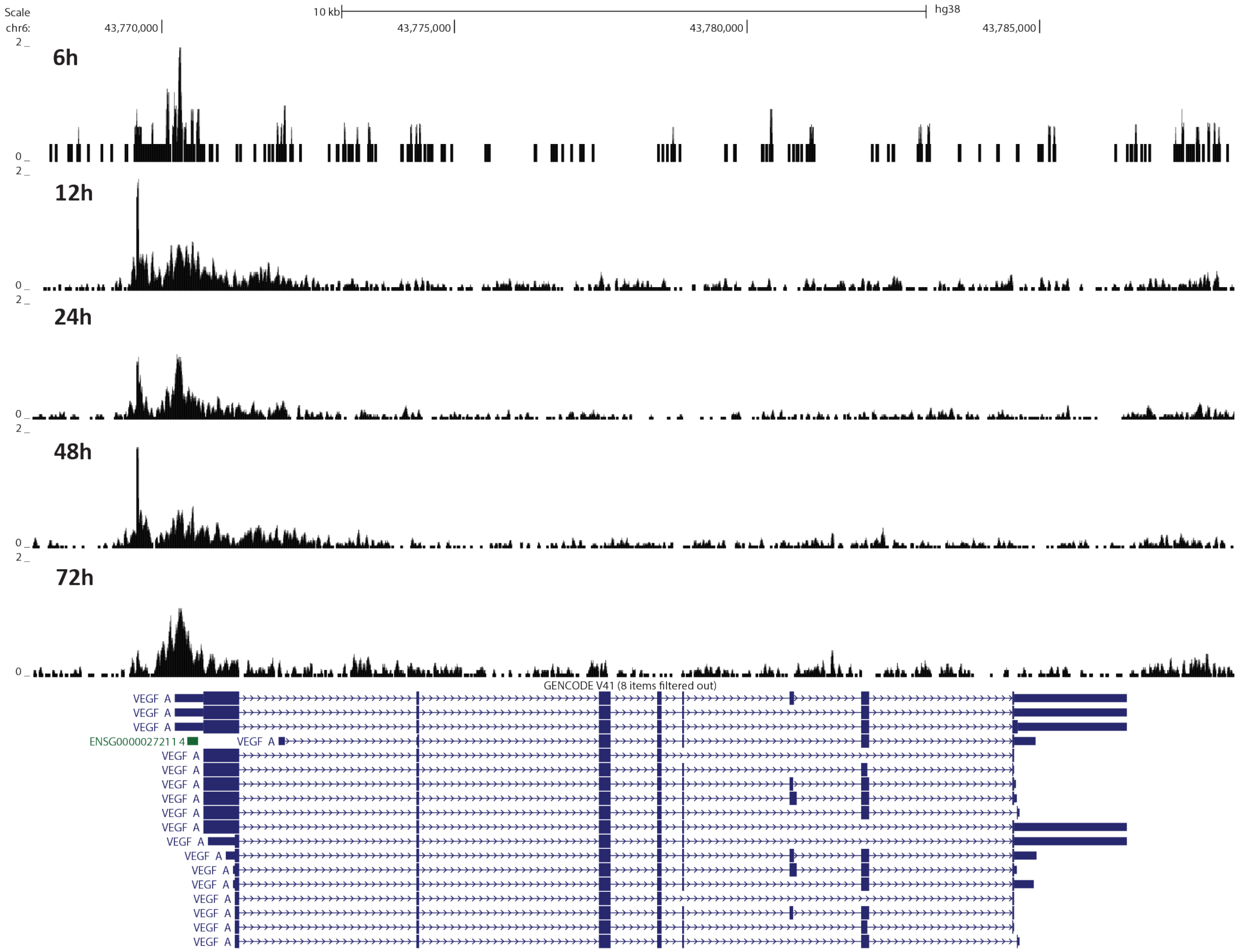

Supplementary Figure 8: Time course of *in vivo* CasKAS signal around the *VEGFA* gene with the VEGFA sgRNA using active Cas9. HEK293 cells were harvested and KAS-seq carried out at the indicated time points after the initiation of the *in vivo* CasKAS experiment.

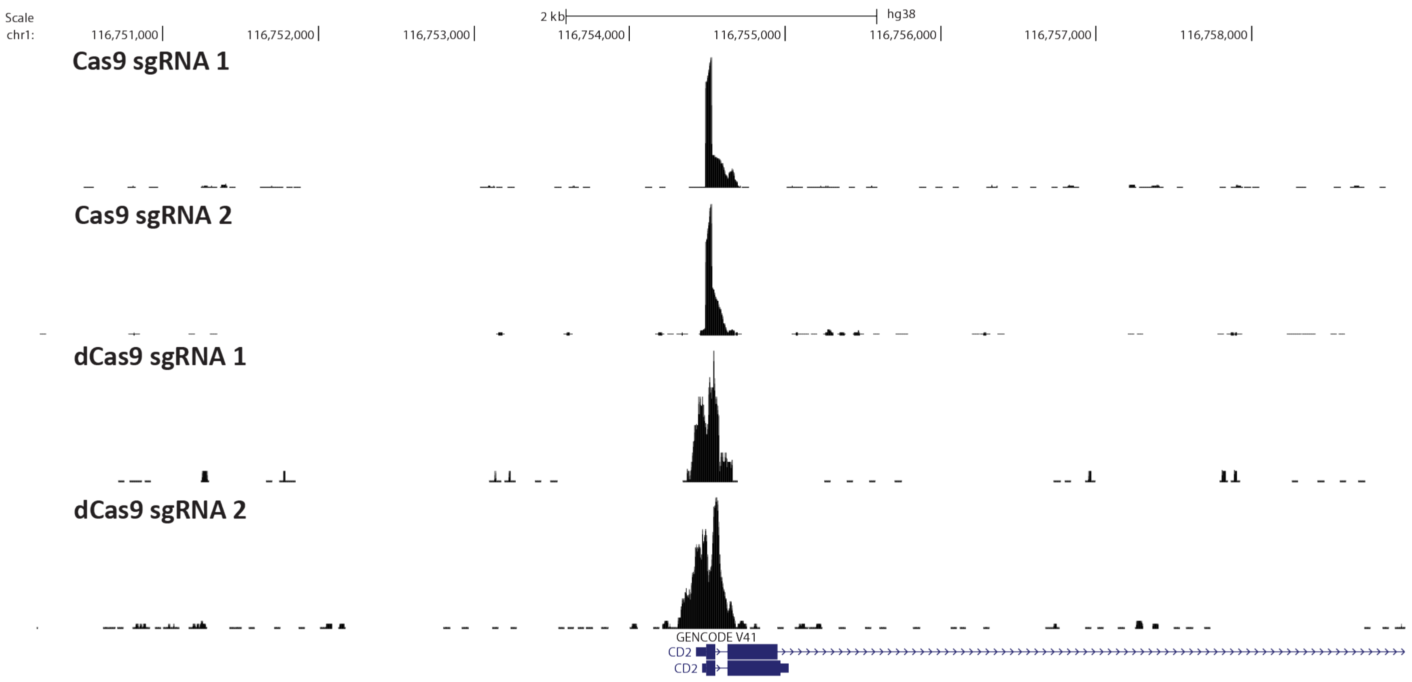

Supplementary Figure 9: CasKAS signal *in vitro* around the *CD2* gene with two different sgRNA targeting the gene.

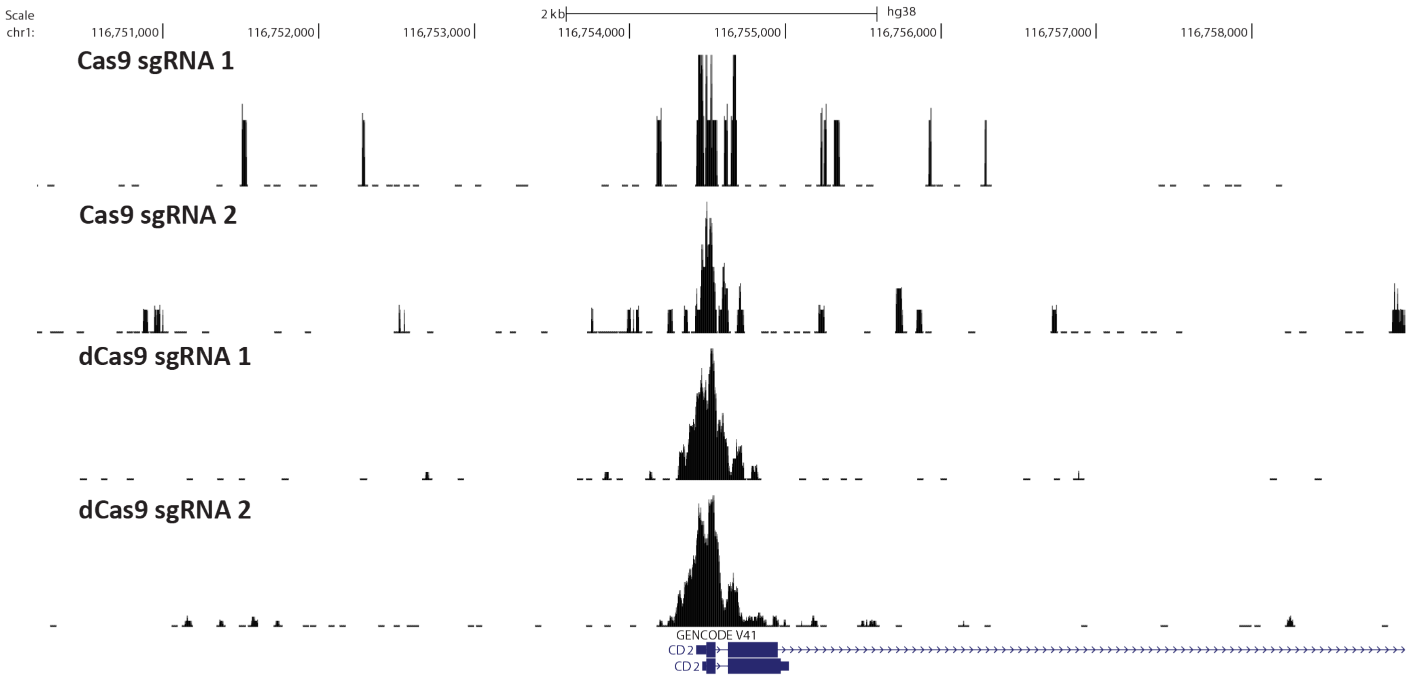

Supplementary Figure 10: CasKAS signal *in vivo* (HEK293 cells, harvested at 48 hours) around the *CD2* gene with two different sgRNA targeting the gene.

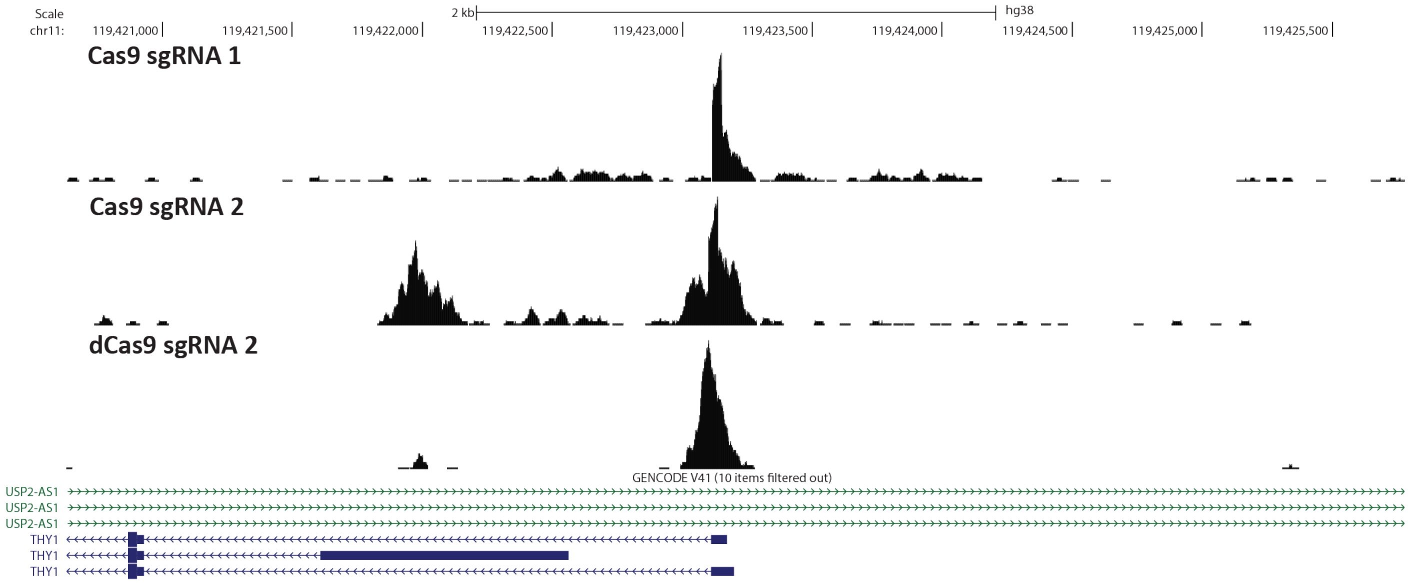

Supplementary Figure 11: CasKAS signal *in vitro* around the *CD90/THY1* gene with two different sgRNA targeting the gene.

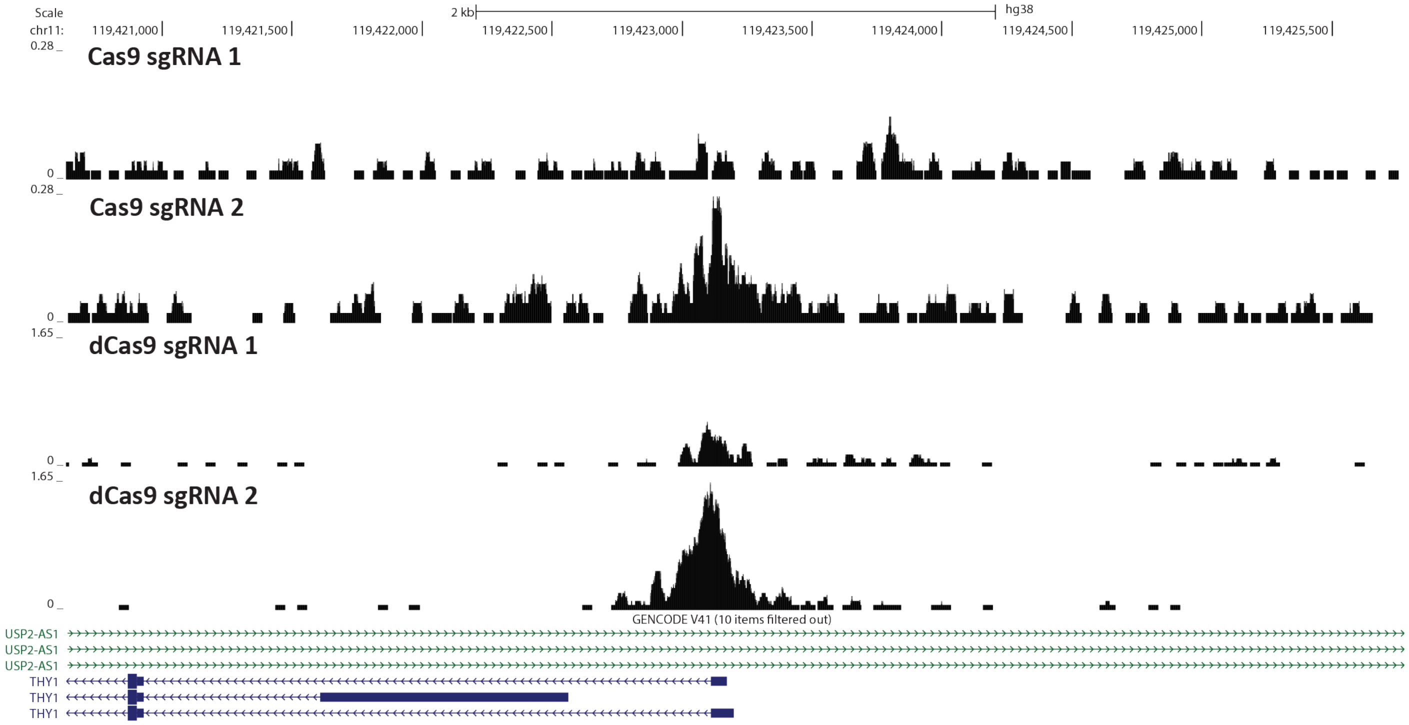

Supplementary Figure 12: CasKAS signal *in vivo* (HEK293 cells, harvested at 48 hours) around the *CD90/THY1* gene with two different sgRNA targeting the gene.

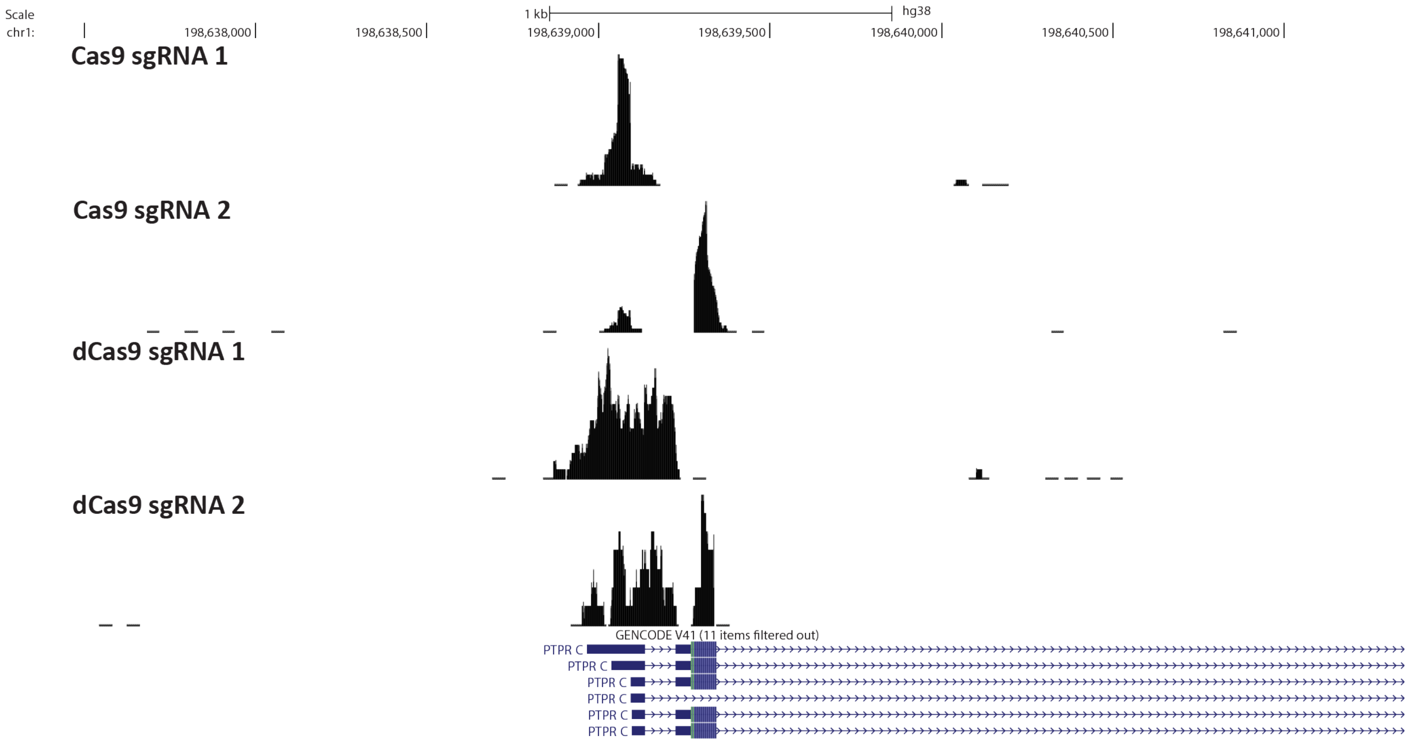

Supplementary Figure 13: CasKAS signal *in vitro* around the *CD45/PTPRC* gene with two different sgRNA targeting the gene.

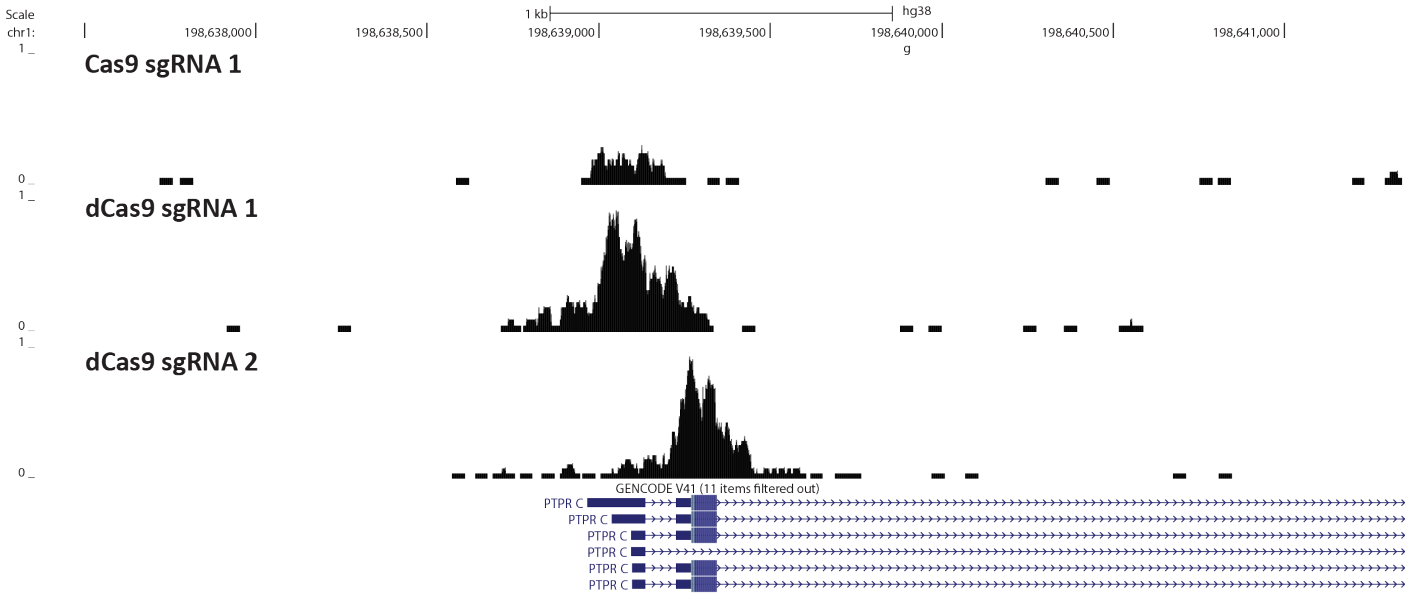

Supplementary Figure 14: CasKAS signal *in vivo* (HEK293 cells, harvested at 48 hours) around the *CD45/PTPRC* gene with two different sgRNA targeting the gene.

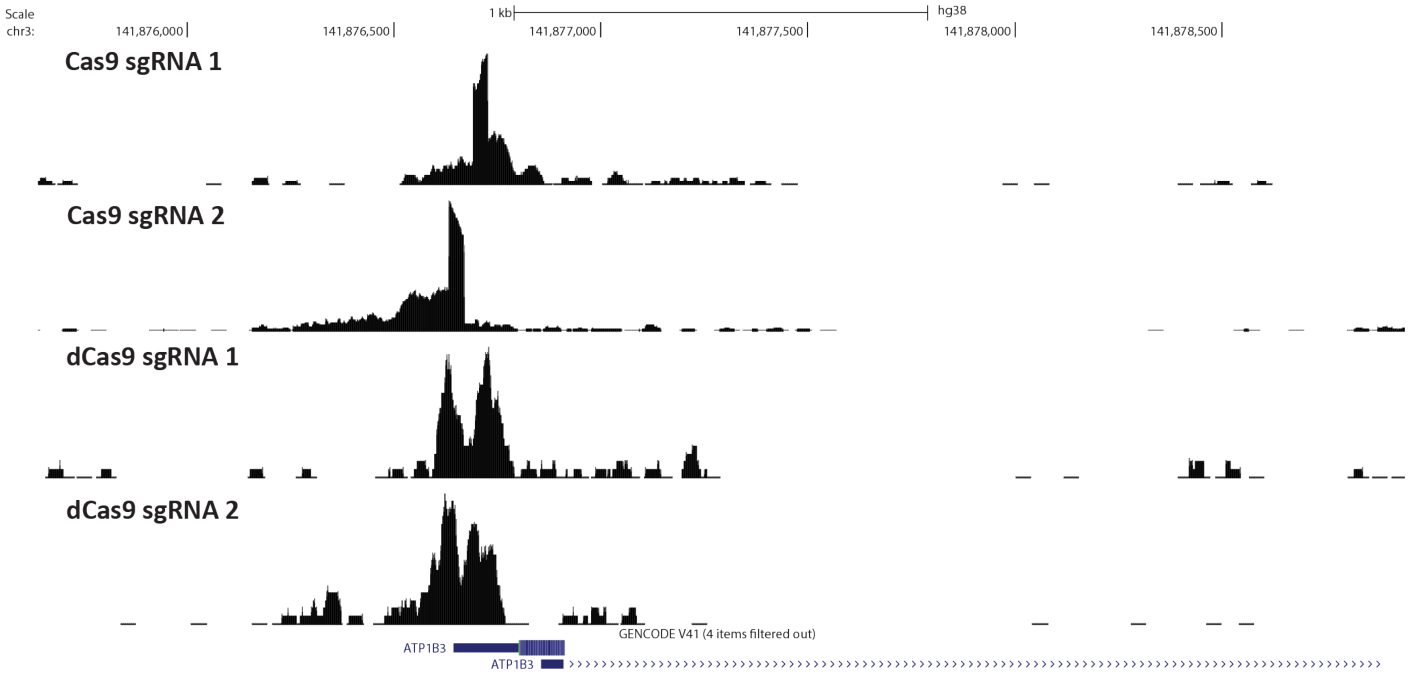

Supplementary Figure 15: CasKAS signal *in vitro* around the *CD298/ATP1B3* gene with two different sgRNA targeting the gene.

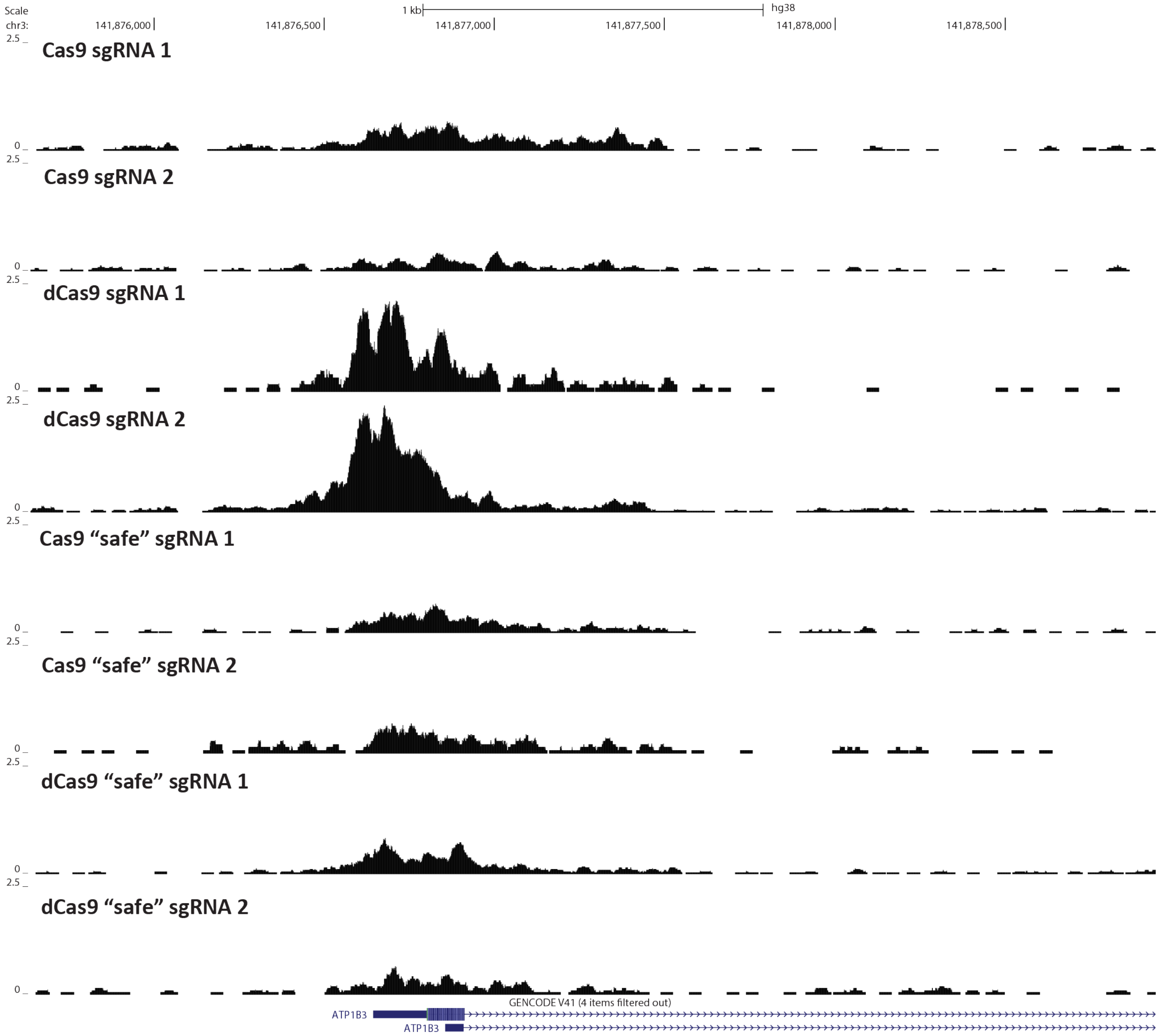

Supplementary Figure 16: CasKAS signal *in vivo* (HEK293 cells, harvested at 48 hours) around the *CD298/ATP1B3* gene with two different sgRNA targeting the gene. Note that in this case the gene displays strong native KAS-seq signal around its promoter in HEK293 cells overlapping the sgRNA targeting sites (see the profiles for "safe" sgRNAs not targeting this locus below). With dCas9 KAS-seq signal above the control levels is observed, but not with with active Cas9, suggesting that the processes generating ssDNA at this locus (e.g. association with RNA polymerases) might be displacing the active Cas9; in contrast, continuous reassociation with dCas9 (as the target sequence is not altered by cleavage) maintains the elevated KAS-seq signal signature.

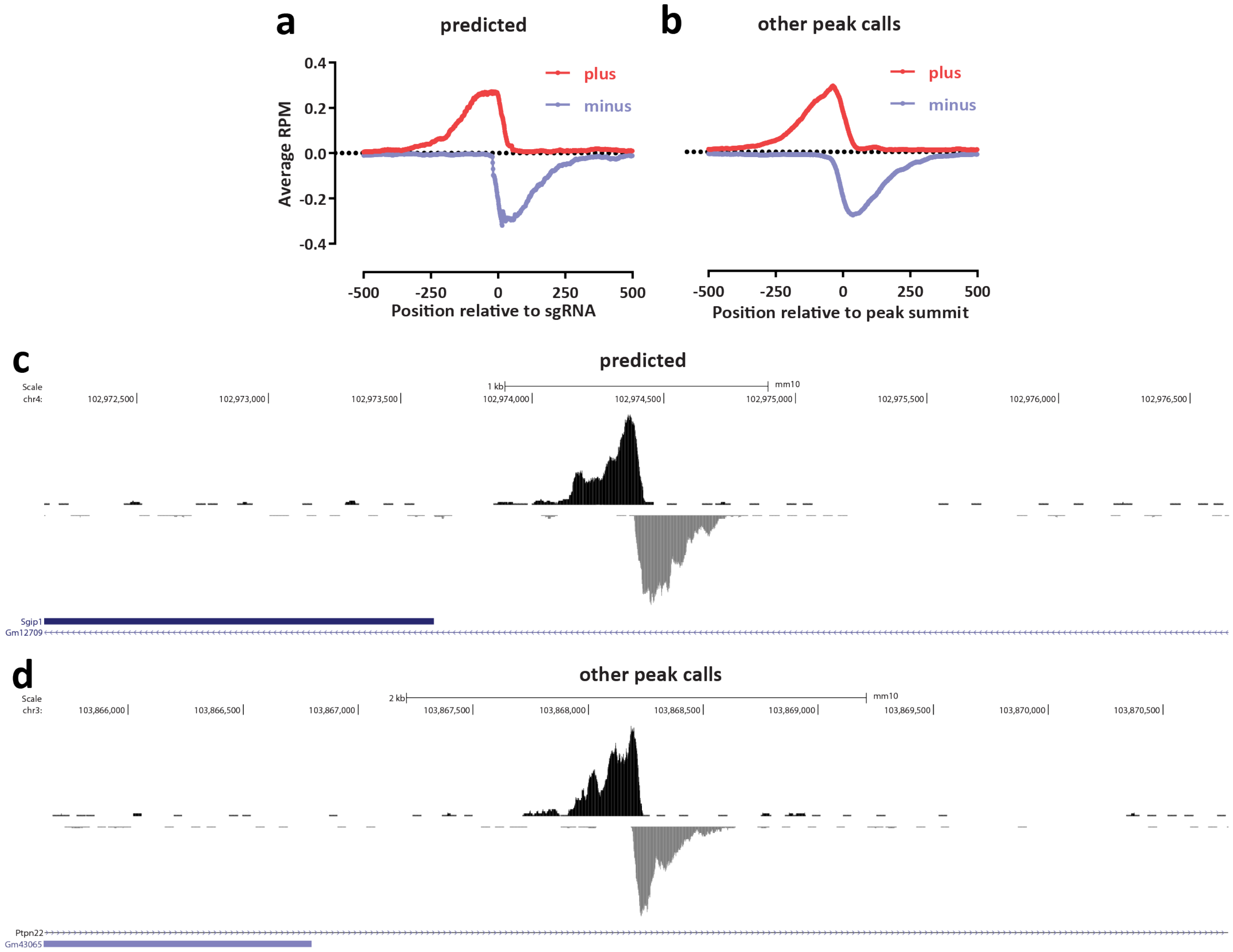

Supplementary Figure 17: CasKAS identifies proper off-target sites that are missed by sgRNA prediction algorithms. Shown is *in vitro* dCas9 CasKAS for the "sgRNA #1" sgRNA. Peaks were called *de novo* using MACS2, then intersected with Cas-OFFinder off-target prediction, and the outersect was manually filtered to exclude obvious artifacts based on peak shape (e.g. arising from repetitive elements in the genome). (a) Aggregate forward- and reverse-strand profiles around off-target sites predicted by Cas-OFFinder (centered on the sgRNA); (b) Aggregate forward- and reverse-strand profiles around sites not predicted by Cas-OFFinder (centered on the MACS2 peak summit); (c) Example UCSC Genome Browser snapshot of a CasKAS read profile around an off-target site predicted by Cas-OFFinder; (c) Example UCSC Genome Browser snapshot of a CasKAS read profile around an off-target site not predicted by Cas-OFFinder. Both predicted and identified through peak calling sites exhibit the expected asymmetric read distribution around a fixed occupancy point (the sgRNA-dCas9 RNP complexed with DNA).

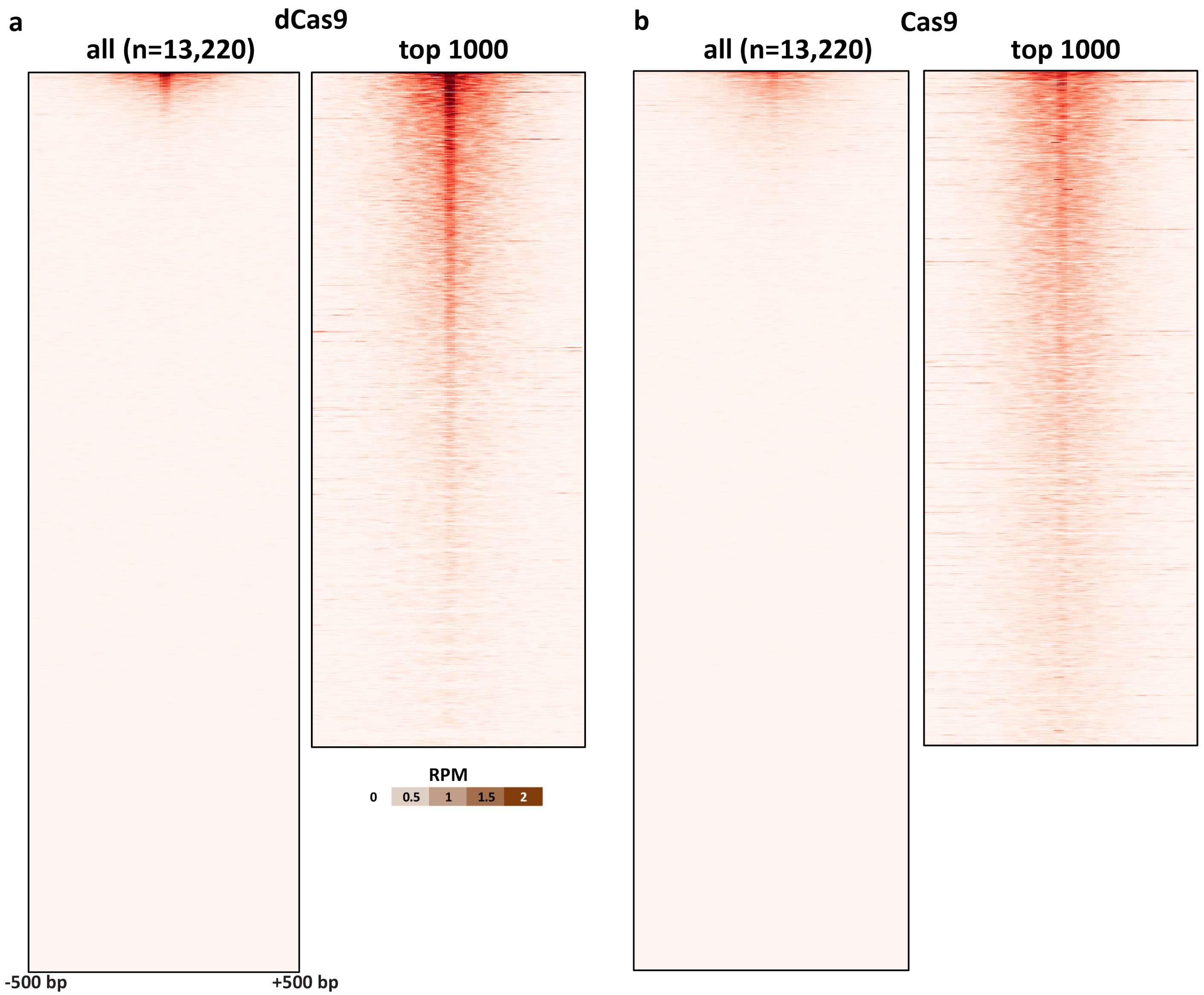

Supplementary Figure 18: ***In vitro*** **dCas9 and Cas9 CasKAS profiles for the "Nanog-sg2" sgRNA**. CasKAS profiles are shown for all off-target sites predicted by Cas-OFFinder as well as for the top 1000 sites (ranked by CasKAS RPM values over the ±500bp region around the sgRNA target site).

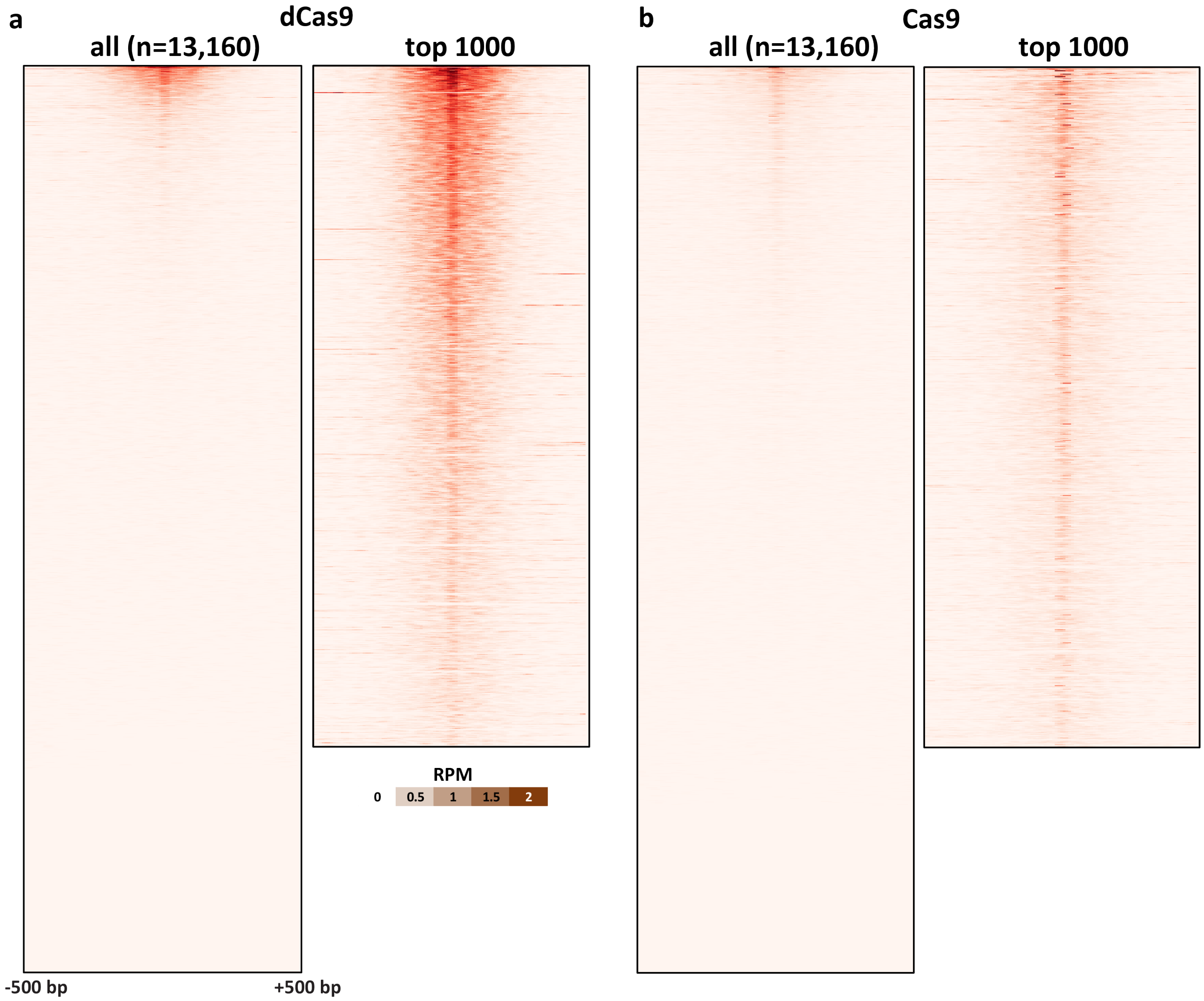

Supplementary Figure 19: *In vitro* dCas9 and Cas9 CasKAS profiles for the "Nanog-sg3" sgRNA. CasKAS profiles are shown for all off-target sites predicted by Cas-OFFinder as well as for the top 1000 sites (ranked by CasKAS RPM values over the ±500bp region around the sgRNA target site).

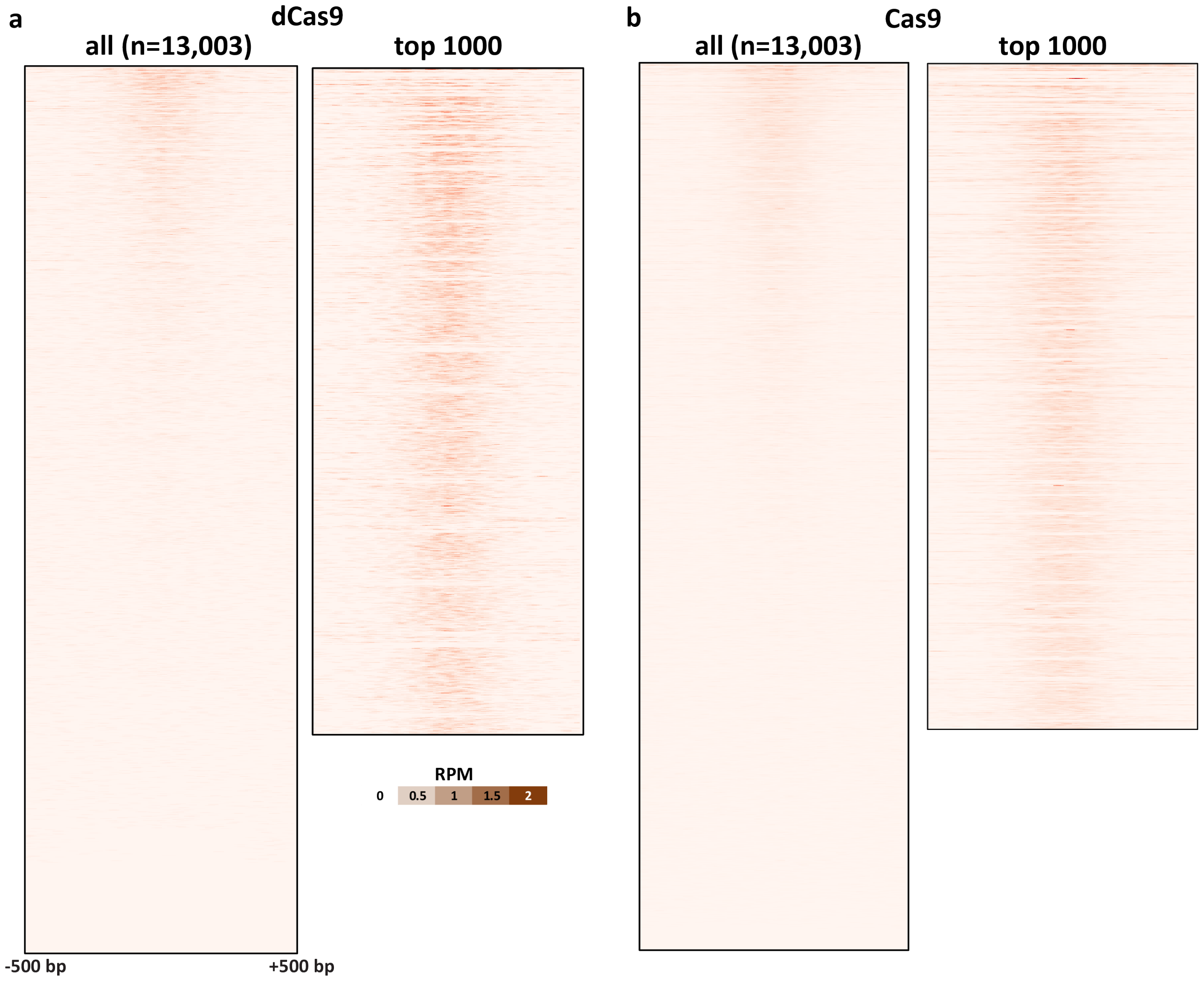

Supplementary Figure 20: *In vitro* dCas9 and Cas9 CasKAS profiles for the "EMX1_Tsai" sgRNA. CasKAS profiles are shown for all off-target sites predicted by Cas-OFFinder as well as for the top 1000 sites (ranked by CasKAS RPM values over the ±500bp region around the sgRNA target site).

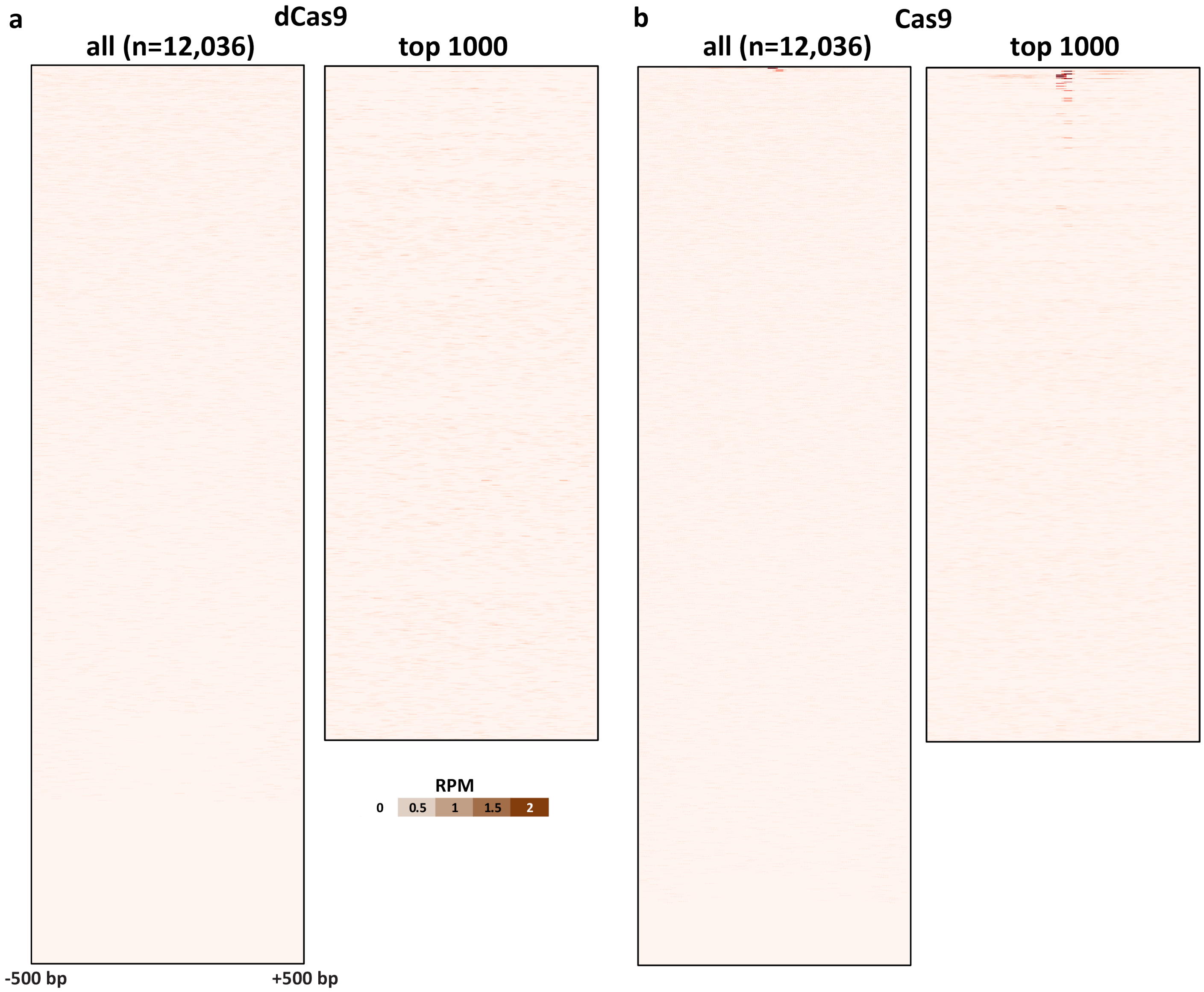

Supplementary Figure 21: ***In vitro*** **dCas9 and Cas9 CasKAS profiles for the "VEGFA-site1" sgRNA**. CasKAS profiles are shown for all off-target sites predicted by Cas-OFFinder as well as for the top 1000 sites (ranked by CasKAS RPM values over the ±500bp region around the sgRNA target site).

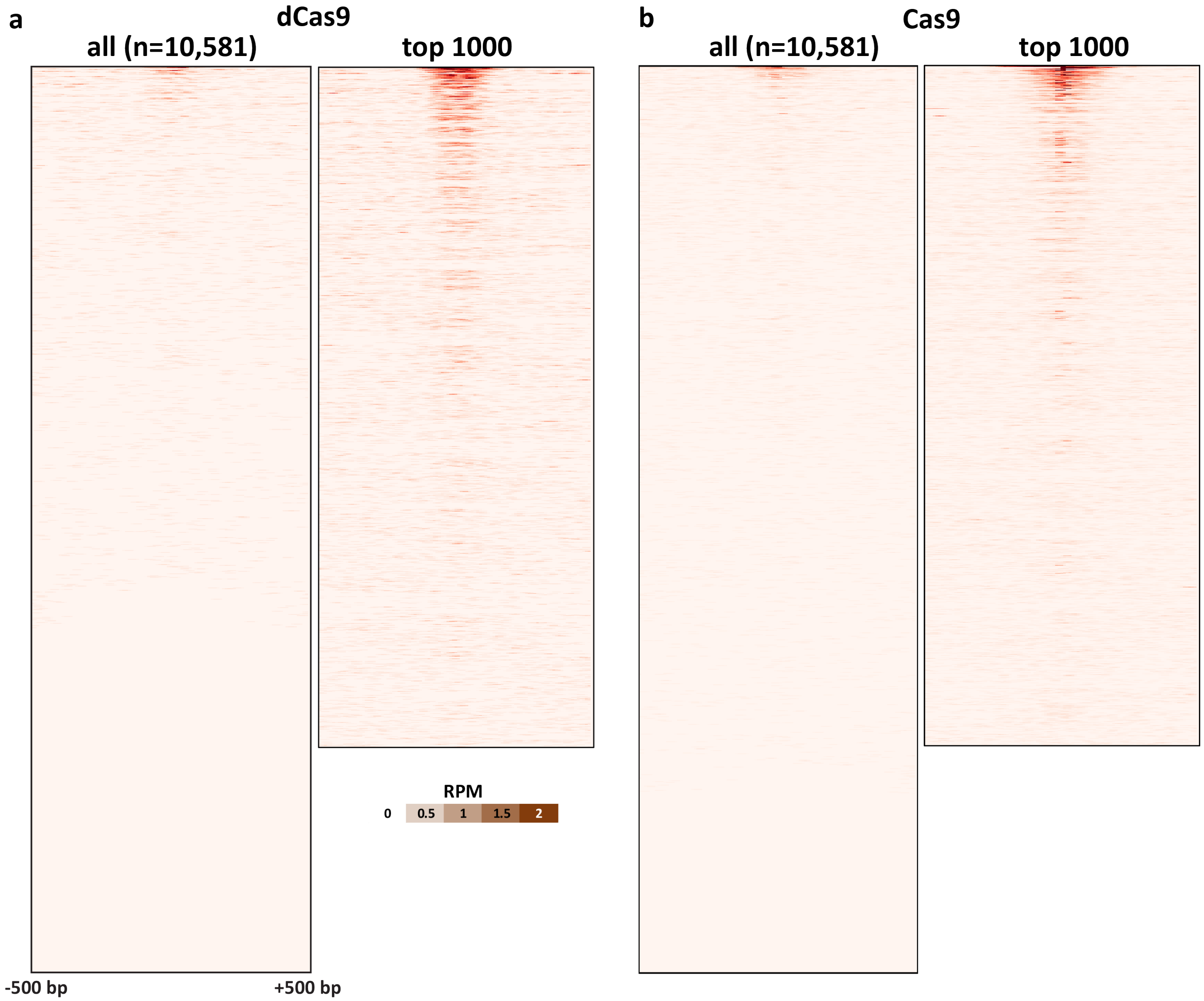

Supplementary Figure 22: ***In vitro*** **dCas9 and Cas9 CasKAS profiles for the "CD2-1" sgRNA**. CasKAS profiles are shown for all off-target sites predicted by Cas-OFFinder as well as for the top 1000 sites (ranked by CasKAS RPM values over the ±500bp region around the sgRNA target site).

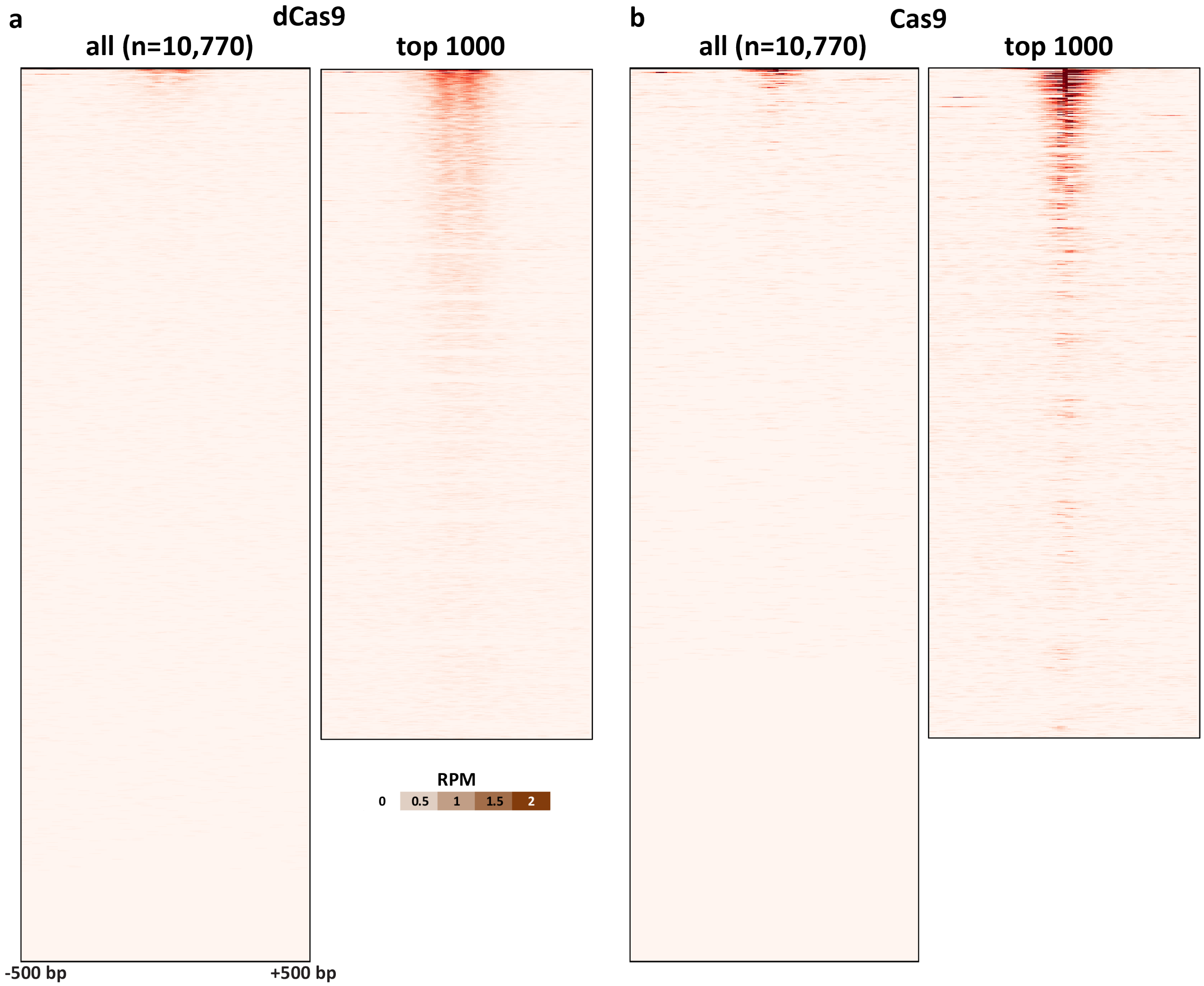

Supplementary Figure 23: ***In vitro*** **dCas9 and Cas9 CasKAS profiles for the "CD2-2" sgRNA**. CasKAS profiles are shown for all off-target sites predicted by Cas-OFFinder as well as for the top 1000 sites (ranked by CasKAS RPM values over the ±500bp region around the sgRNA target site).

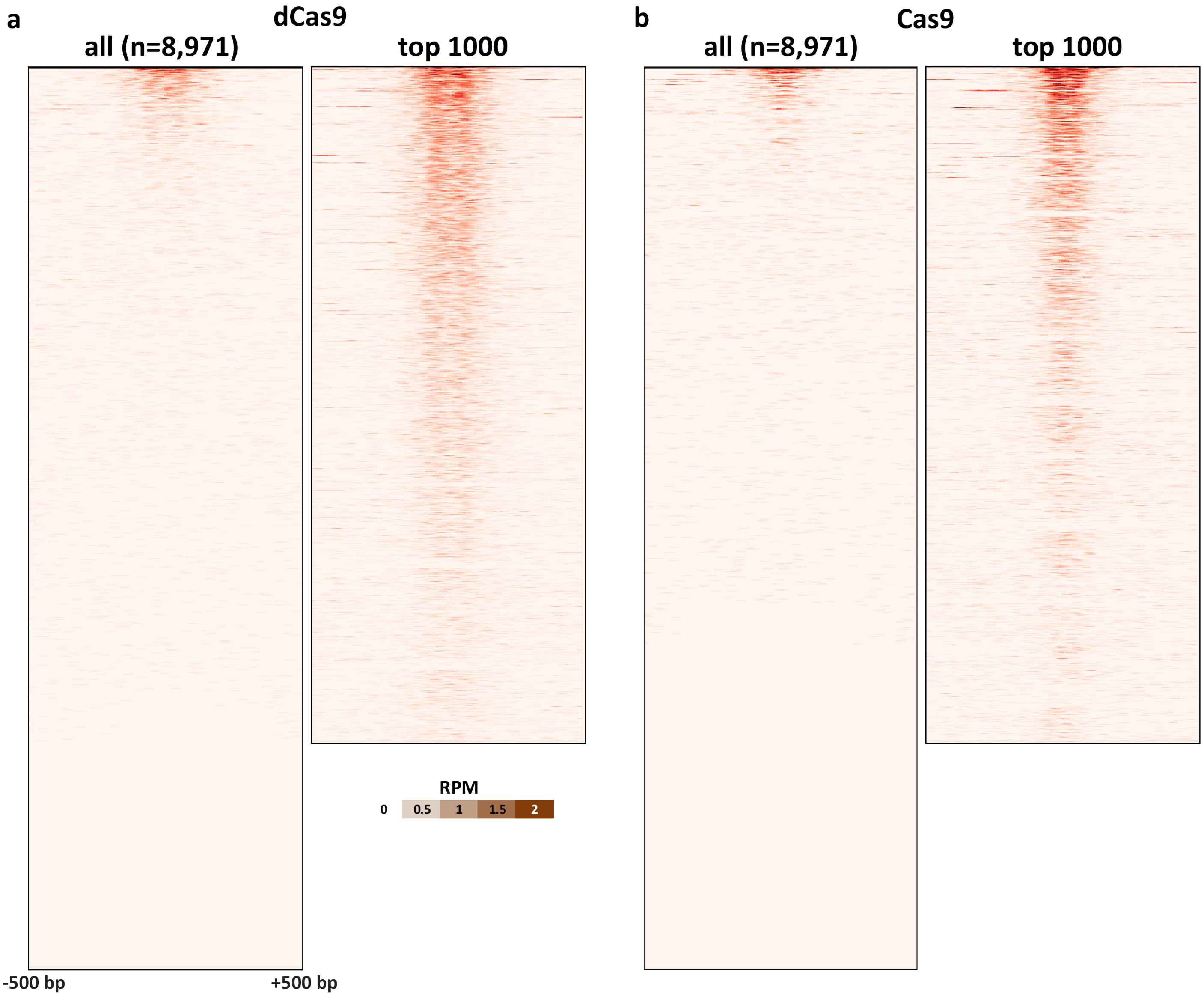

Supplementary Figure 24: *In vitro* dCas9 and Cas9 CasKAS profiles for the "CD45-1" sgRNA. CasKAS profiles are shown for all off-target sites predicted by Cas-OFFinder as well as for the top 1000 sites (ranked by CasKAS RPM values over the ±500bp region around the sgRNA target site).

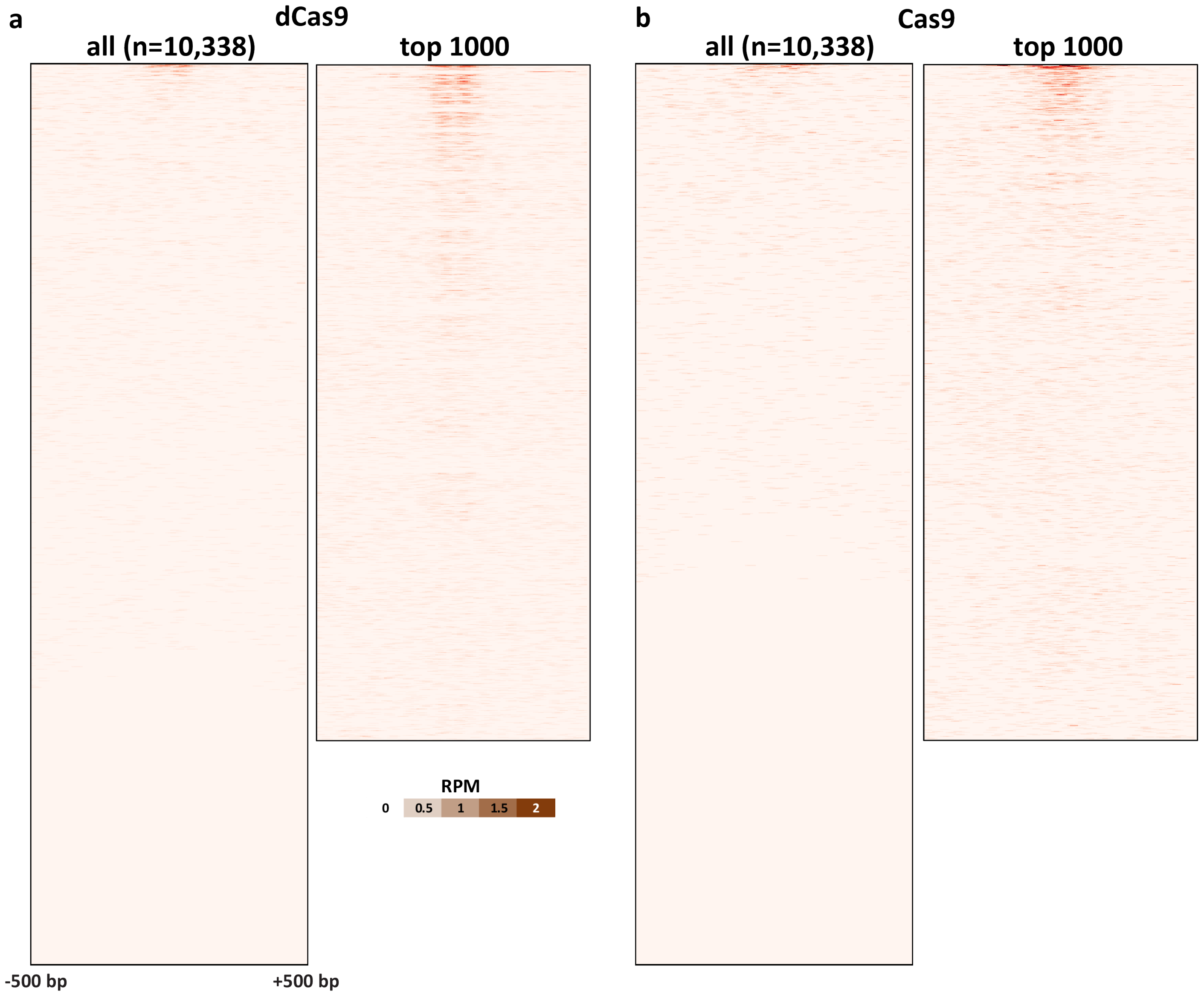

Supplementary Figure 25: ***In vitro*** **dCas9 and Cas9 CasKAS profiles for the "CD45-2" sgRNA**. CasKAS profiles are shown for all off-target sites predicted by Cas-OFFinder as well as for the top 1000 sites (ranked by CasKAS RPM values over the ±500bp region around the sgRNA target site).

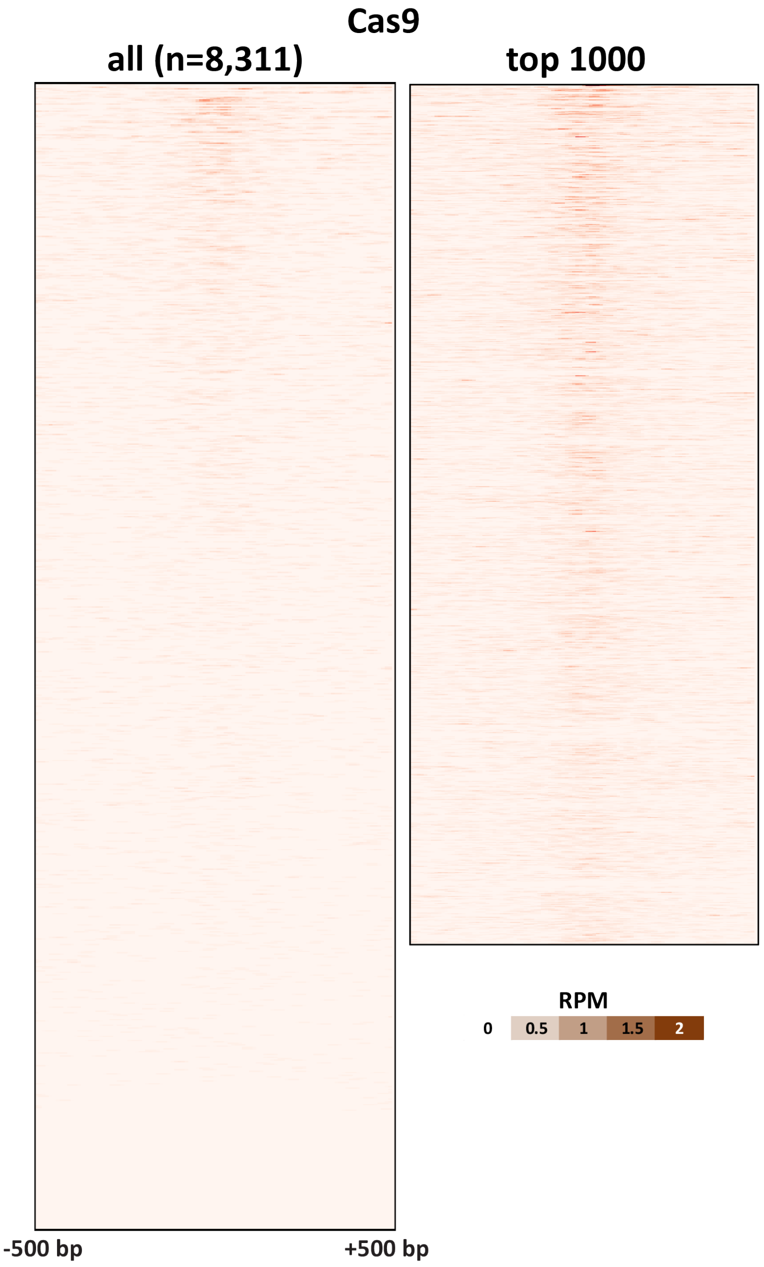

Supplementary Figure 26: ***In vitro*** **Cas9 CasKAS profiles for the "CD90-1" sgRNA**. CasKAS profiles are shown for all off-target sites predicted by Cas-OFFinder as well as for the top 1000 sites (ranked by CasKAS RPM values over the ±500bp region around the sgRNA target site).

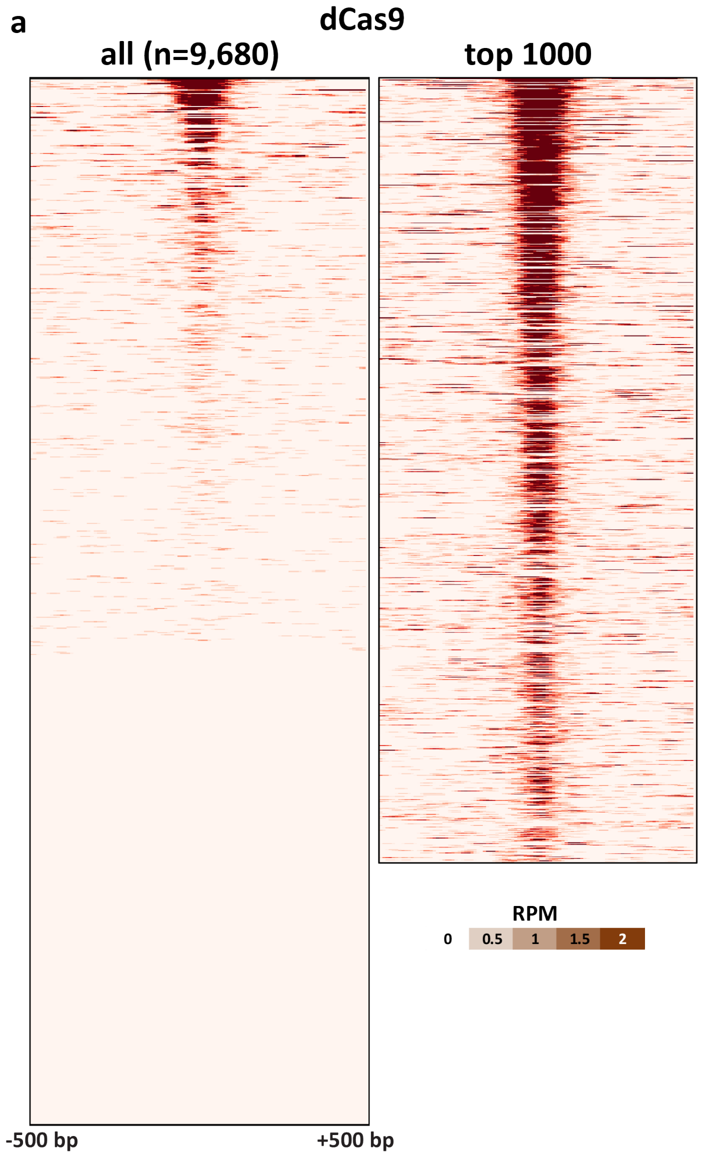

Supplementary Figure 27: ***In vitro*** **dCas9 and Cas9 CasKAS profiles for the "CD90-2" sgRNA**. CasKAS profiles are shown for all off-target sites predicted by Cas-OFFinder as well as for the top 1000 sites (ranked by CasKAS RPM values over the ±500bp region around the sgRNA target site).

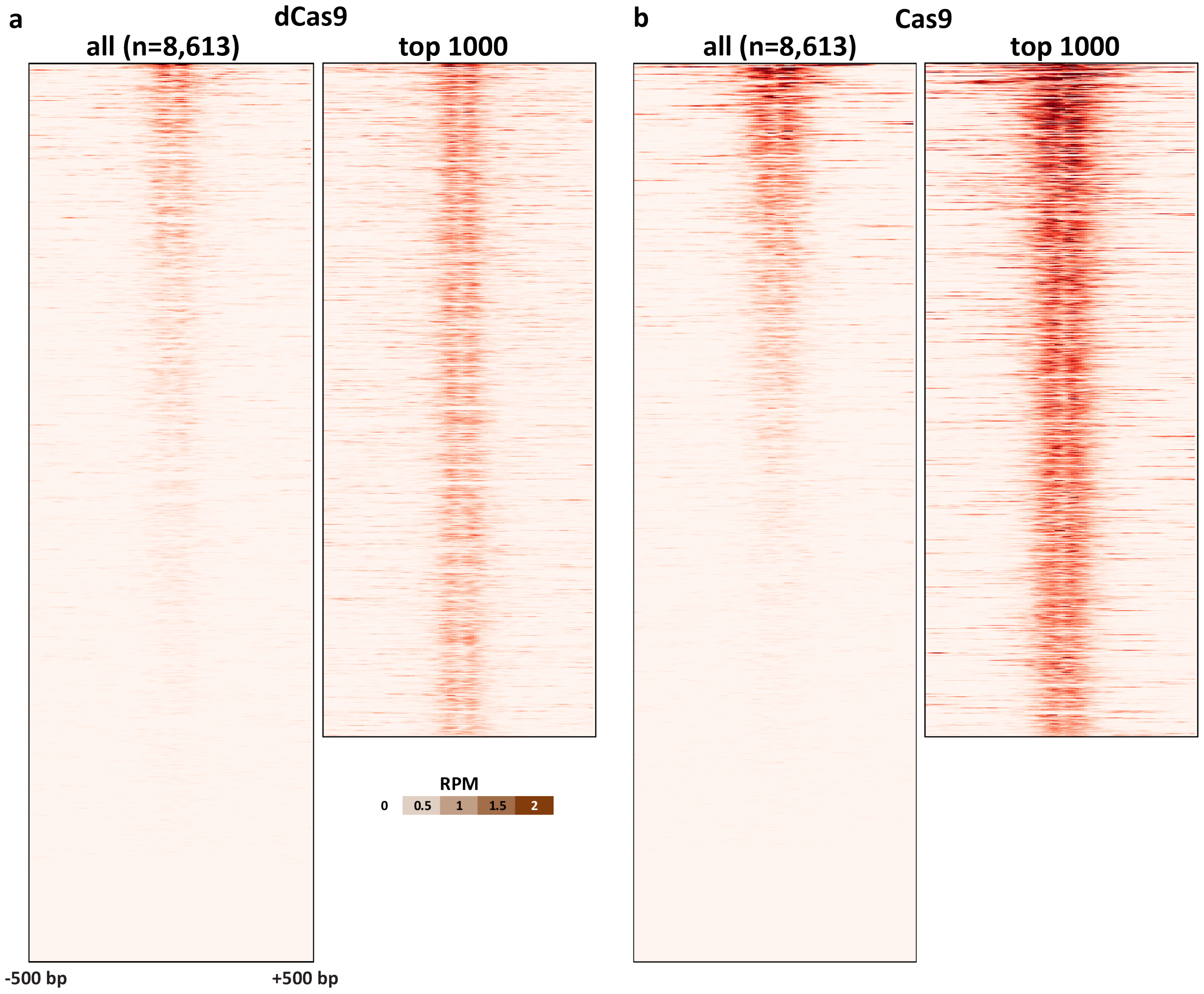

Supplementary Figure 28: *In vitro* dCas9 and Cas9 CasKAS profiles for the "CD298-1" sgRNA. CasKAS profiles are shown for all off-target sites predicted by Cas-OFFinder as well as for the top 1000 sites (ranked by CasKAS RPM values over the ±500bp region around the sgRNA target site).

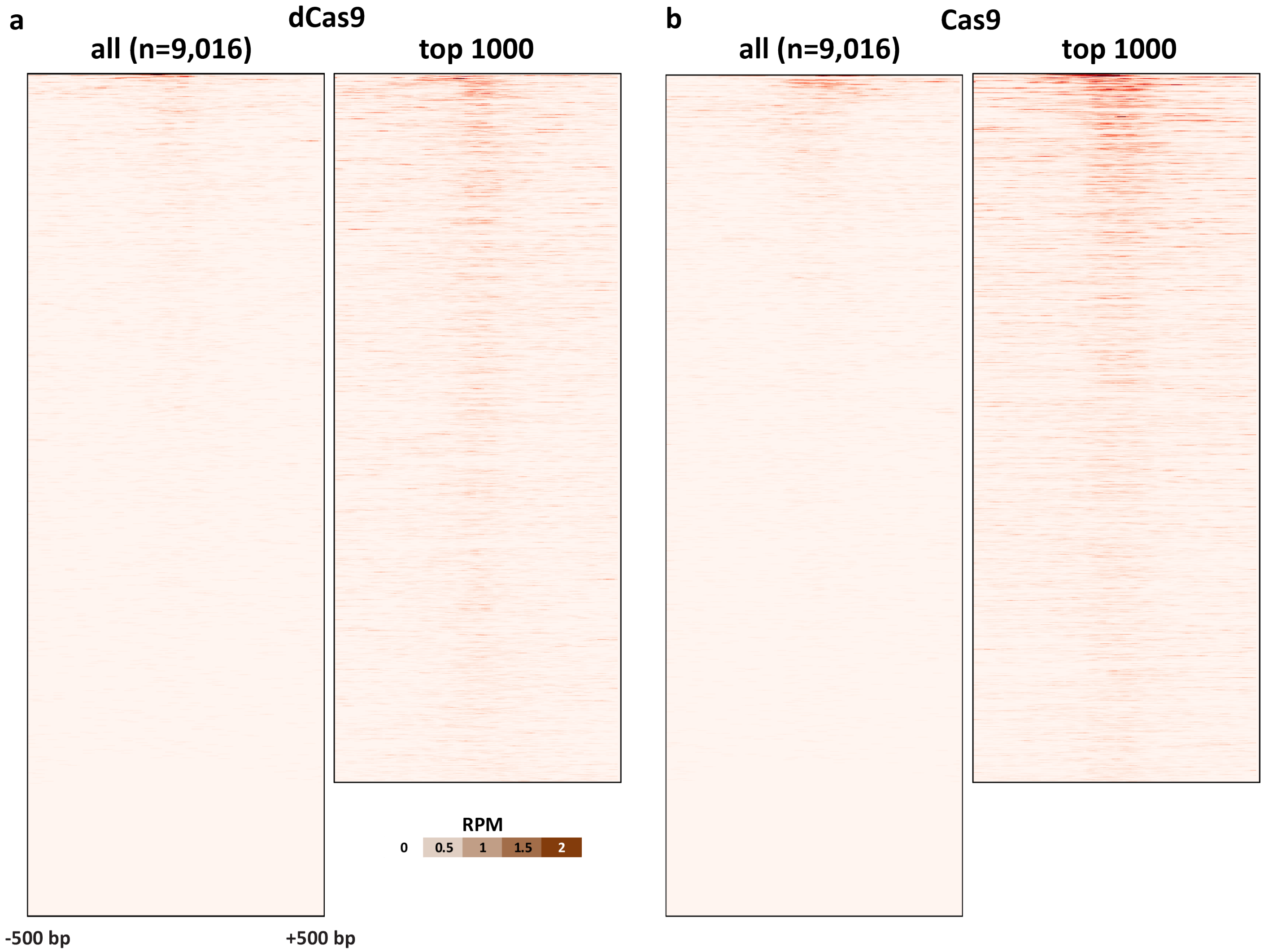

Supplementary Figure 29: ***In vitro*** **dCas9 and Cas9 CasKAS profiles for the "CD298-2" sgRNA**. CasKAS profiles are shown for all off-target sites predicted by Cas-OFFinder as well as for the top 1000 sites (ranked by CasKAS RPM values over the ±500bp region around the sgRNA target site).

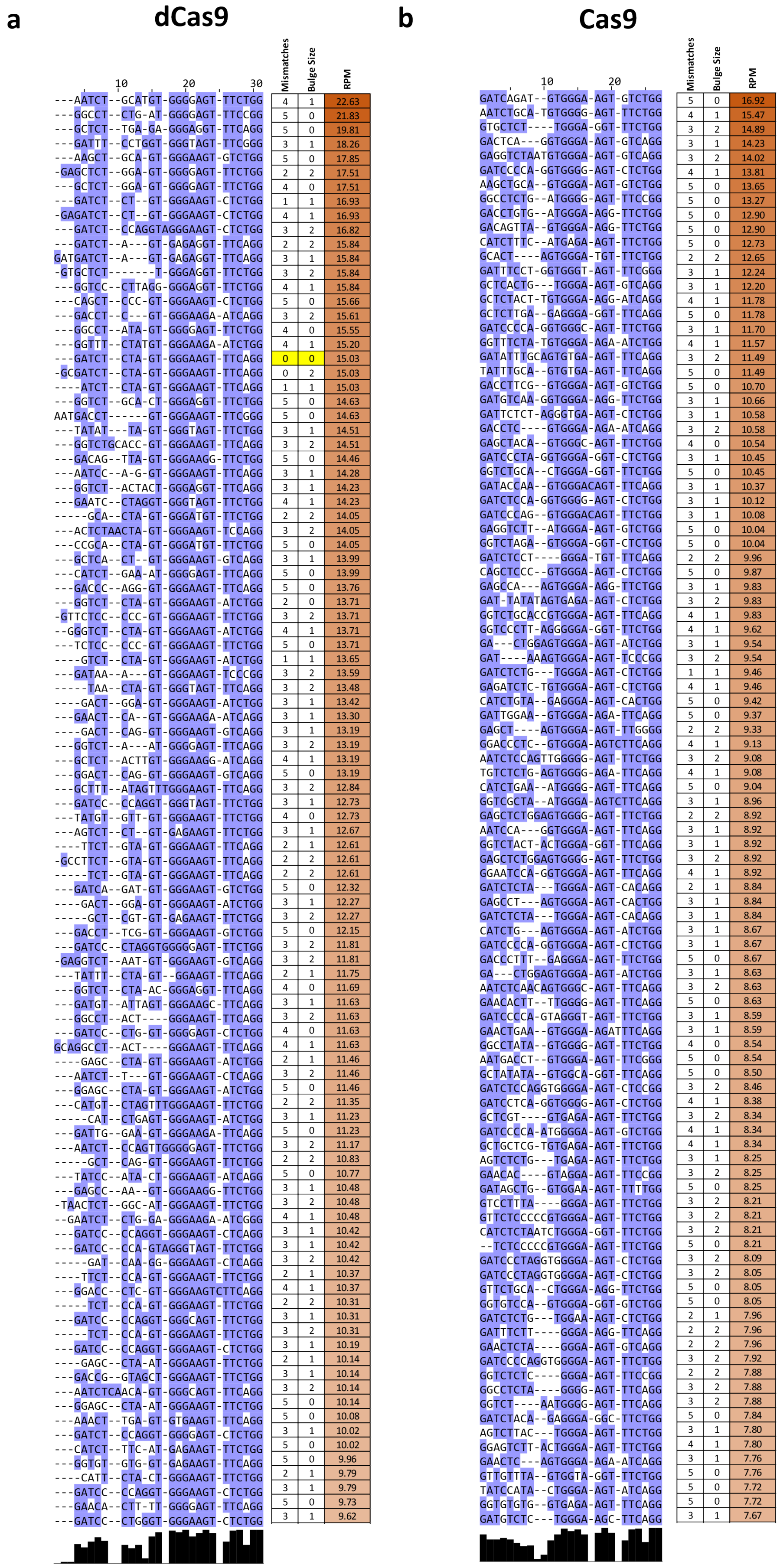

Supplementary Figure 30: Multiple sequence alignment of off-target sites identified by *in vitro* dCas9 and Cas9 CasKAS for the "Nanog-sg2" sgRNA. Shown are the top 100 off-target sites as predicted by Cas-OFFinder and ranked by CasKAS signal. The on-target site (if within the top 100) is highlighted in yellow. The black bars on the bottom indicate the degree of sequence conservation for a given position within the multiple sequence alignment.

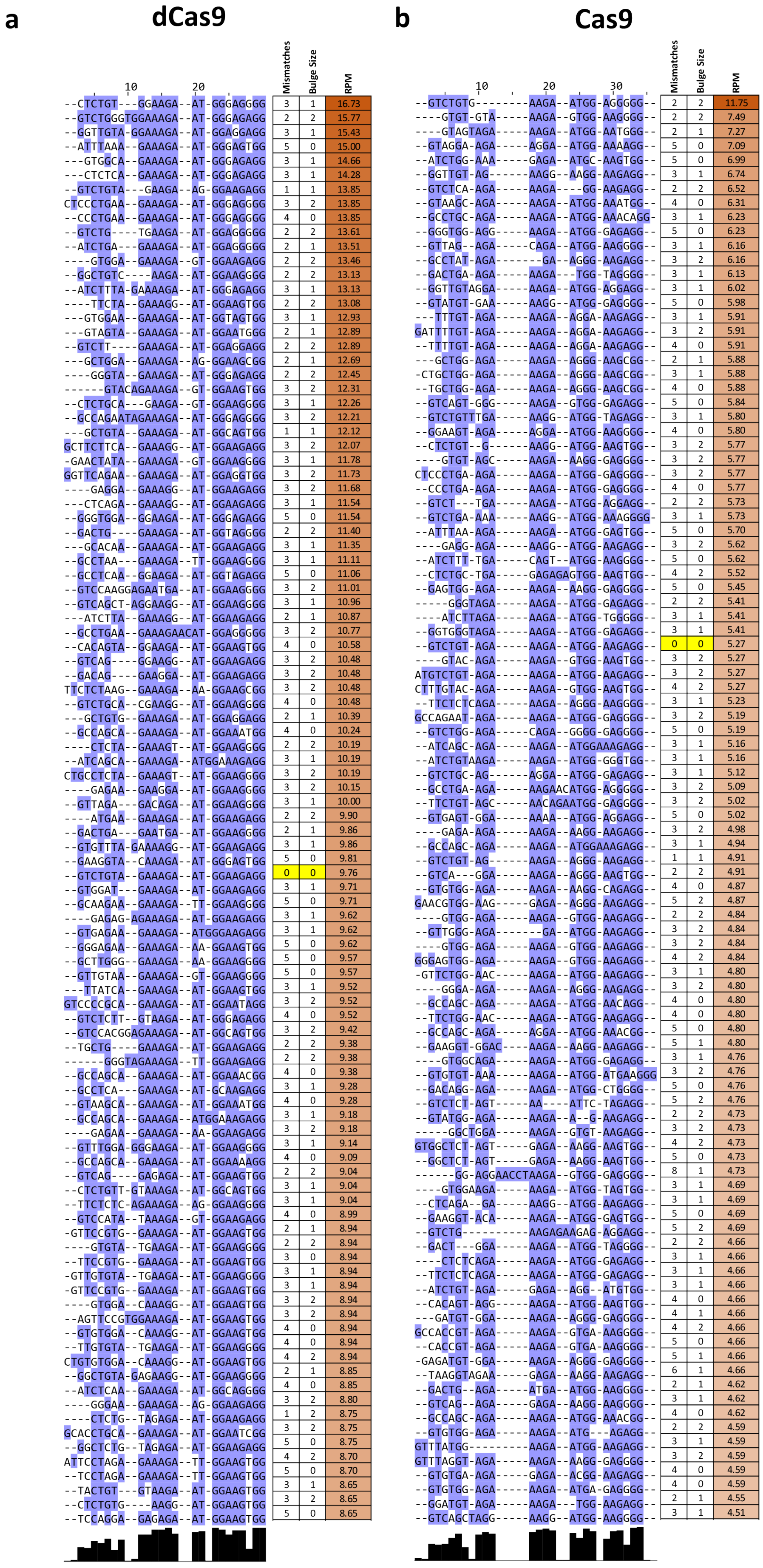

Supplementary Figure 31: Multiple sequence alignment of off-target sites identified by *in vitro* dCas9 and Cas9 CasKAS for the "Nanog-sg3" sgRNA. Shown are the top 100 off-target sites as predicted by Cas-OFFinder and ranked by CasKAS signal. The on-target site (if within the top 100) is highlighted in yellow. The black bars on the bottom indicate the degree of sequence conservation for a given position within the multiple sequence alignment.

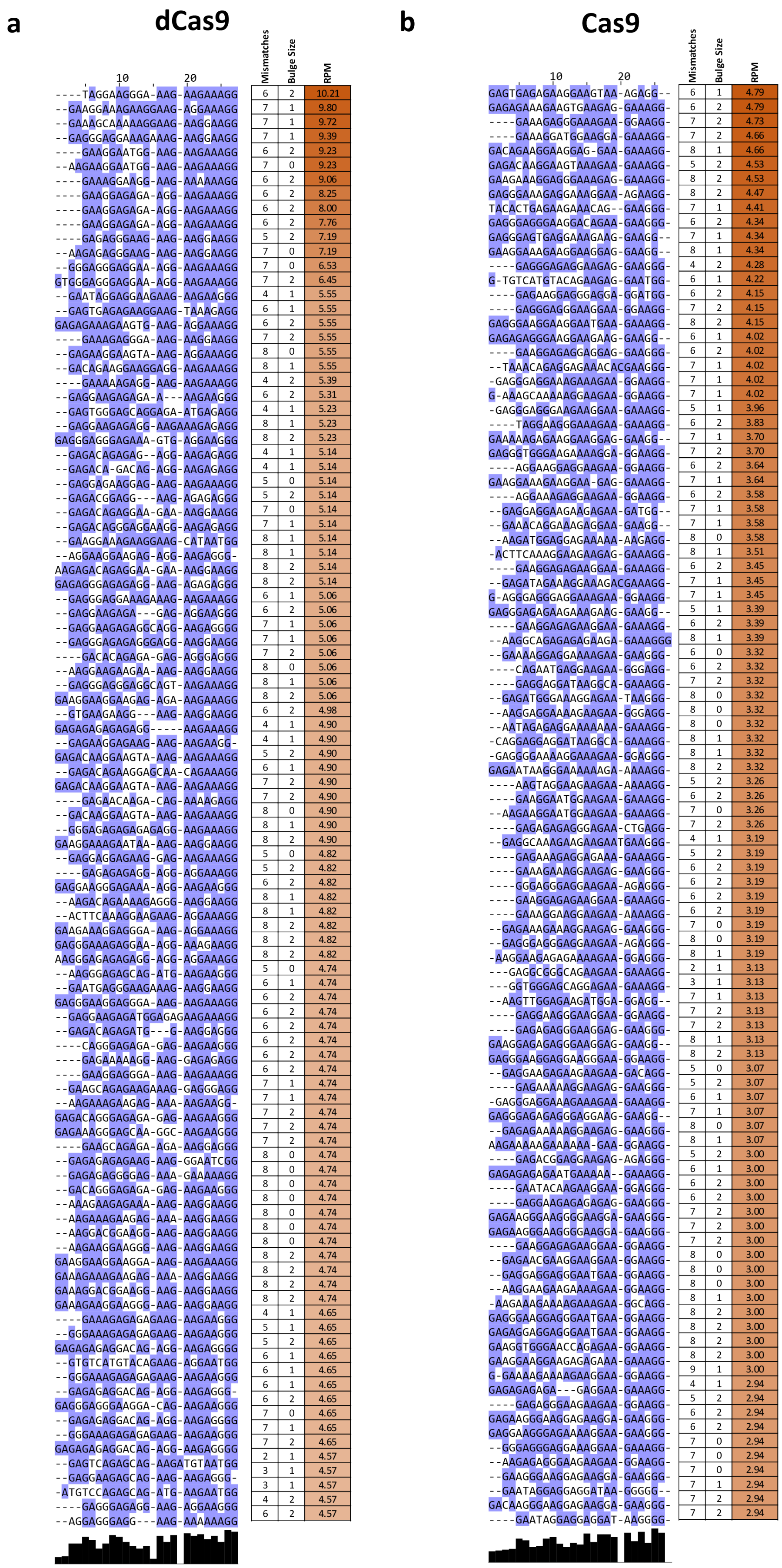

Supplementary Figure 32: Multiple sequence alignment of off-target sites identified by *in vitro* dCas9 and Cas9 CasKAS for the "EMX1_Tsai" sgRNA. Shown are the top 100 off-target sites as predicted by Cas-OFFinder and ranked by CasKAS signal. The on-target site (if within the top 100) is highlighted in yellow. The black bars on the bottom indicate the degree of sequence conservation for a given position within the multiple sequence alignment.

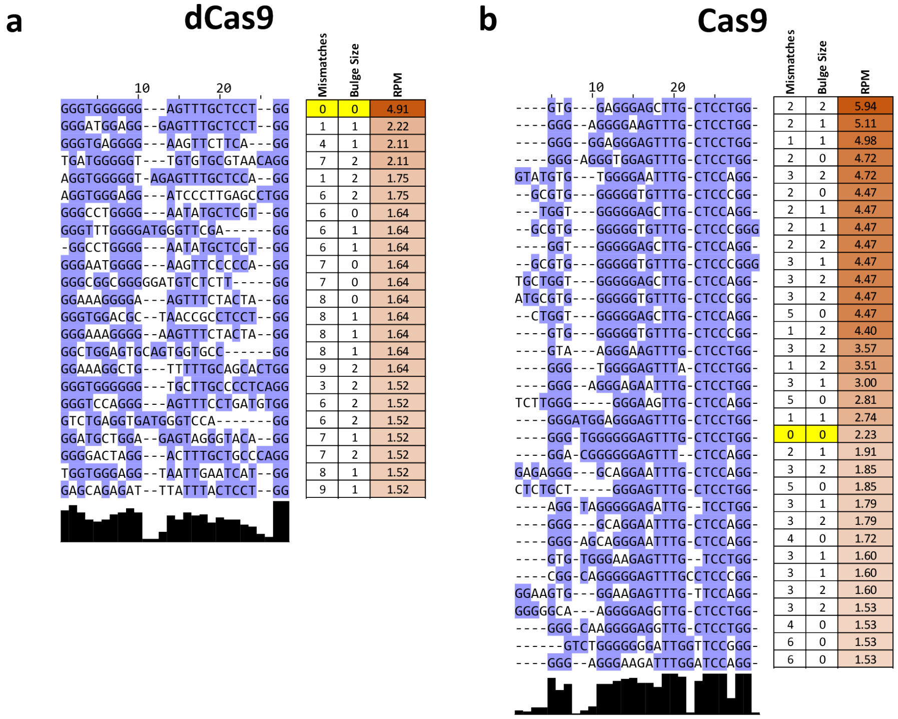

Supplementary Figure 33: Multiple sequence alignment of off-target sites identified by *in vitro* dCas9 and Cas9 CasKAS for the "VEGFA-site1" sgRNA. Shown are the all target sites with RPM ≥ 1.5 as predicted by Cas-OFFinder and ranked by CasKAS signal. The on-target site (if within the top 100) is highlighted in yellow. The black bars on the bottom indicate the degree of sequence conservation for a given position within the multiple sequence alignment.

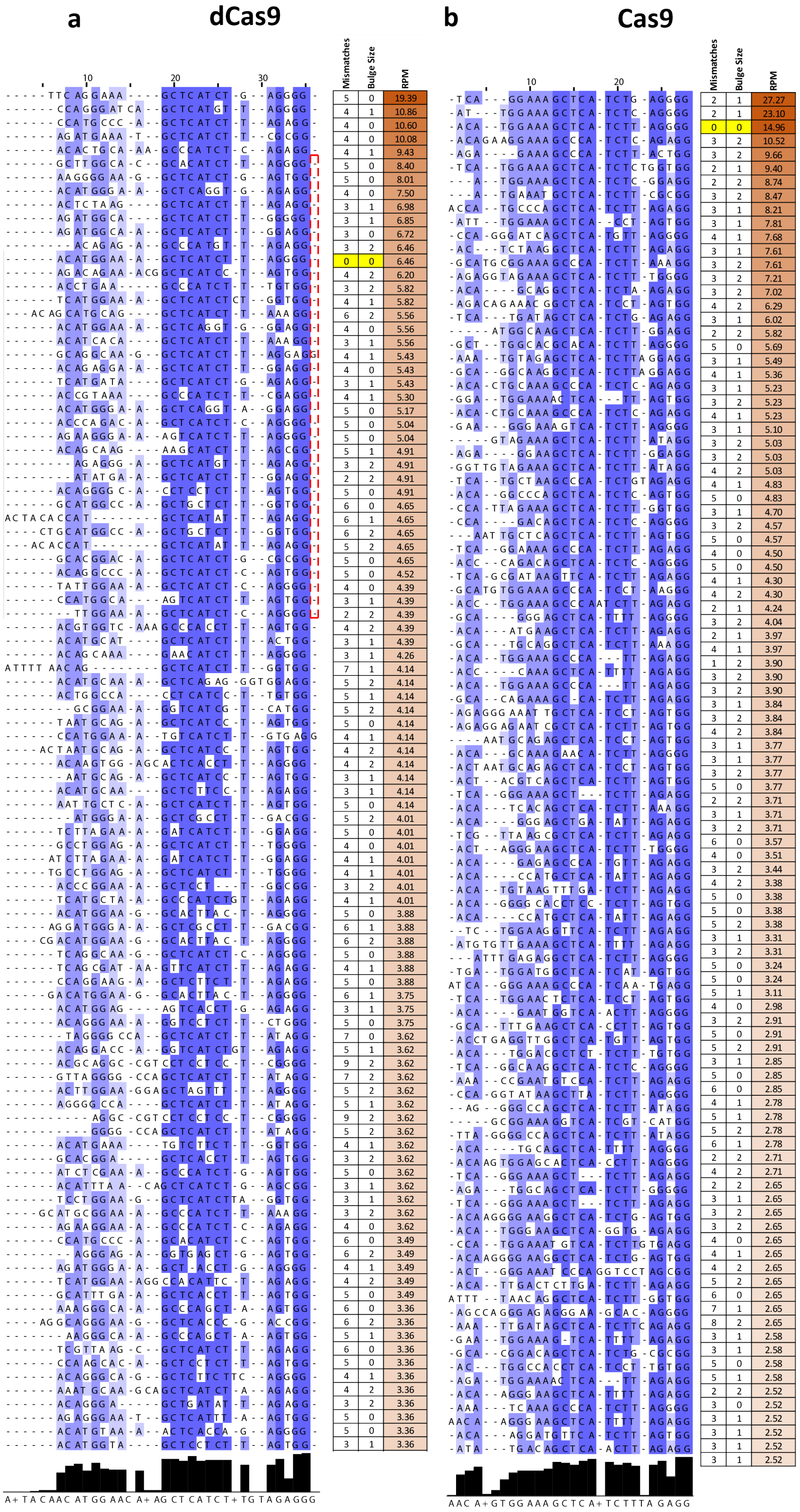

Supplementary Figure 34: Multiple sequence alignment of off-target sites identified by *in vitro* dCas9 and Cas9 CasKAS for the "CD2-1" sgRNA. Shown are the top 100 off-target sites as predicted by Cas-OFFinder and ranked by CasKAS signal. The on-target site (if within the top 100) is highlighted in yellow. The black bars on the bottom indicate the degree of sequence conservation for a given position within the multiple sequence alignment.

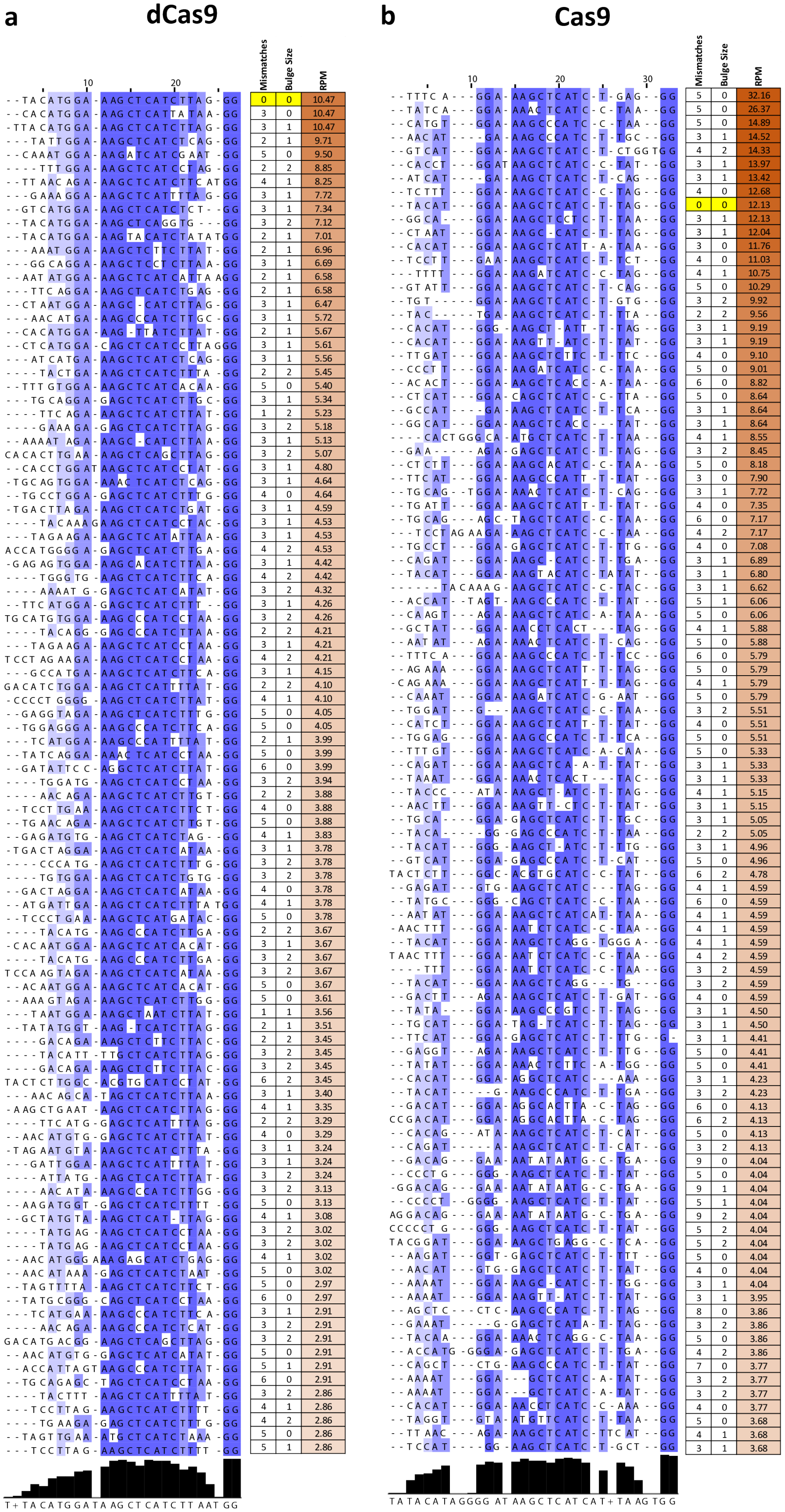

Supplementary Figure 35: Multiple sequence alignment of off-target sites identified by *in vitro* dCas9 and Cas9 CasKAS for the "CD-2" sgRNA. Shown are the top 100 off-target sites as predicted by Cas-OFFinder and ranked by CasKAS signal. The on-target site (if within the top 100) is highlighted in yellow. The black bars on the bottom indicate the degree of sequence conservation for a given position within the multiple sequence alignment.

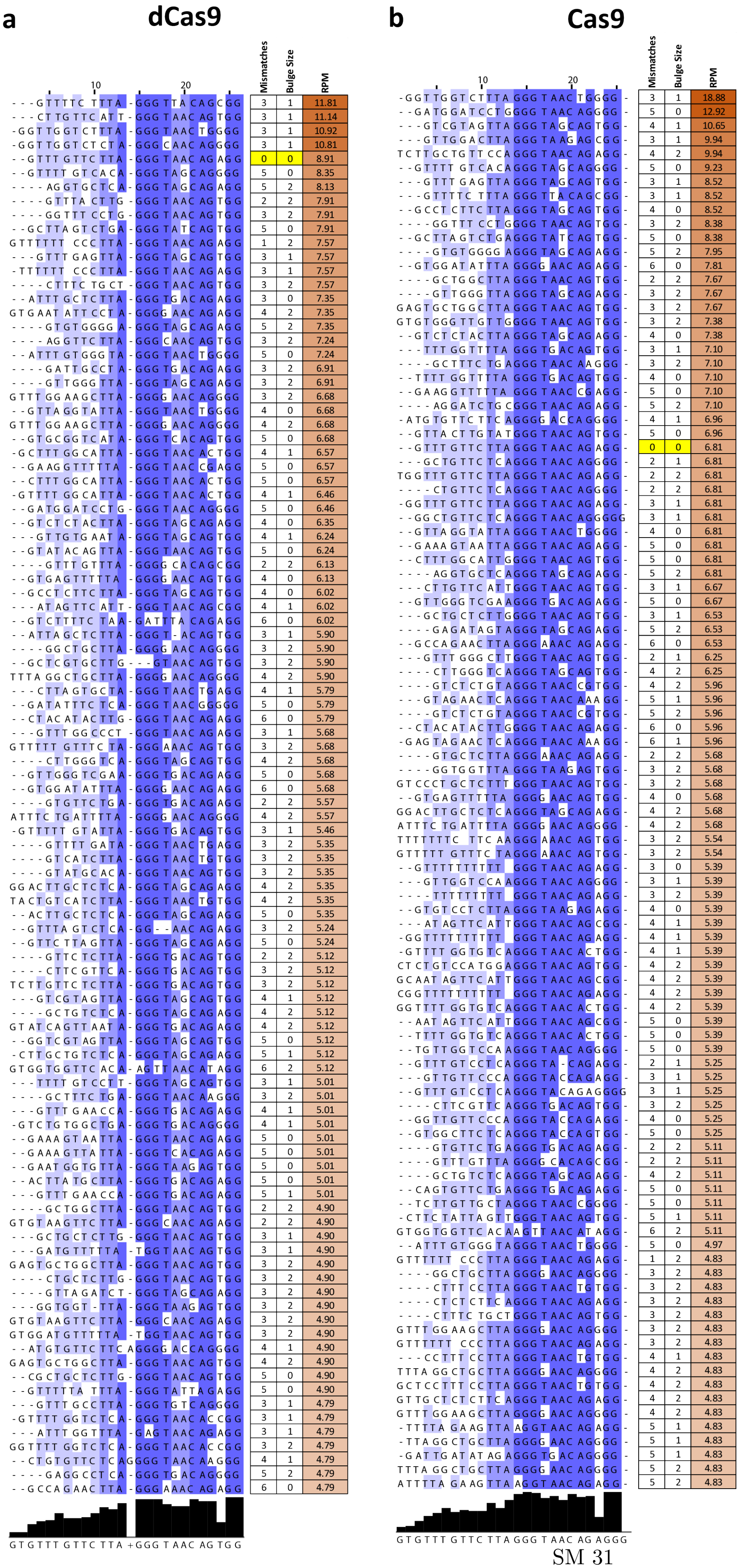

Supplementary Figure 36: Multiple sequence alignment of off-target sites identified by *in vitro* dCas9 and Cas9 CasKAS for the "CD45-1" sgRNA. Shown are the top 100 off-target sites as predicted by Cas-OFFinder and ranked by CasKAS signal. The on-target site (if within the top 100) is highlighted in yellow. The black bars on the bottom indicate the degree of sequence conservation for a given position within the multiple sequence alignment.

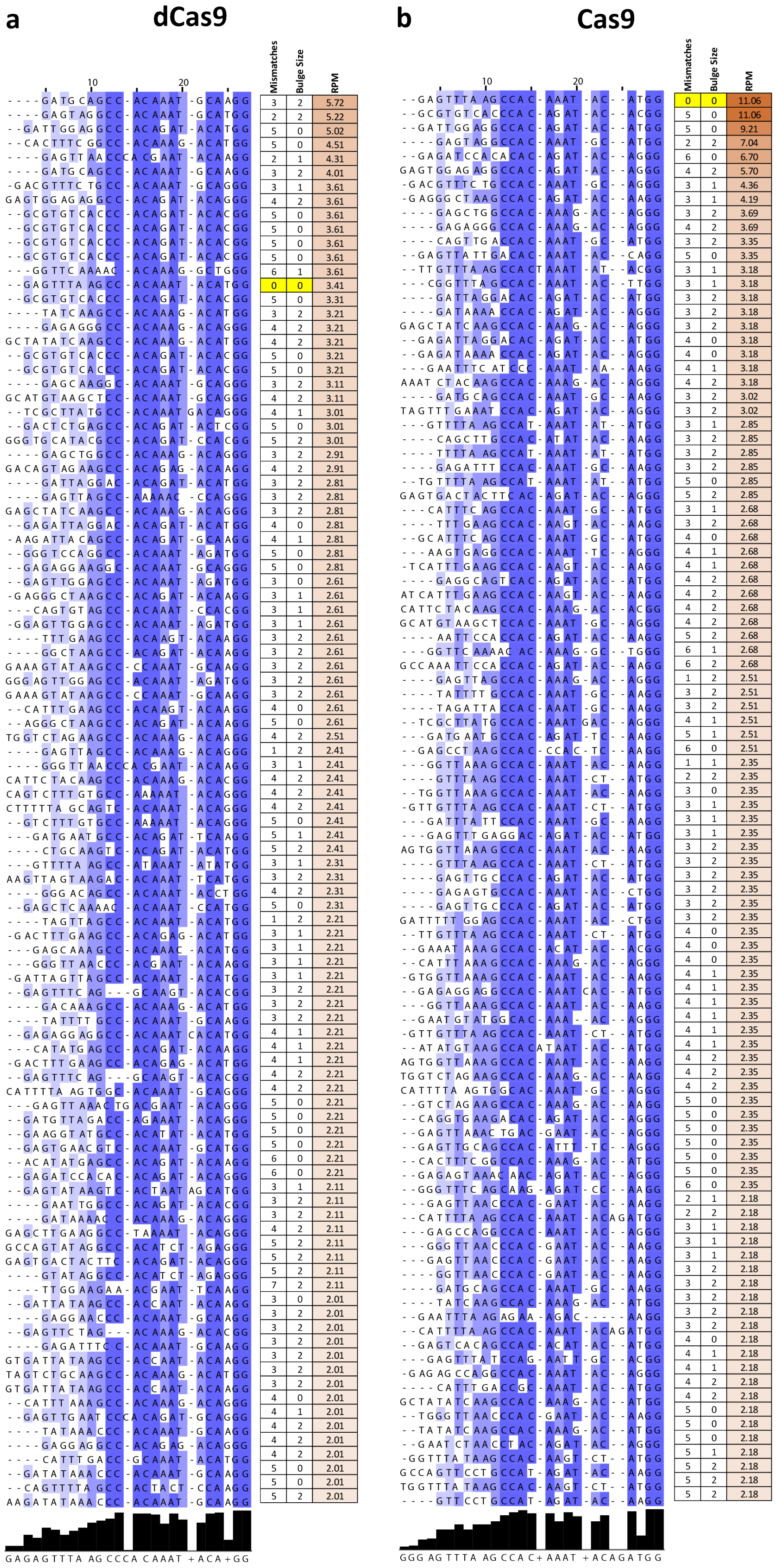

Supplementary Figure 37: Multiple sequence alignment of off-target sites identified by *in vitro* dCas9 and Cas9 CasKAS for the "CD45-2" sgRNA. Shown are the top 100 off-target sites as predicted by Cas-OFFinder and ranked by CasKAS signal. The on-target site (if within the top 100) is highlighted in yellow. The black bars on the bottom indicate the degree of sequence conservation for a given position within the multiple sequence alignment.

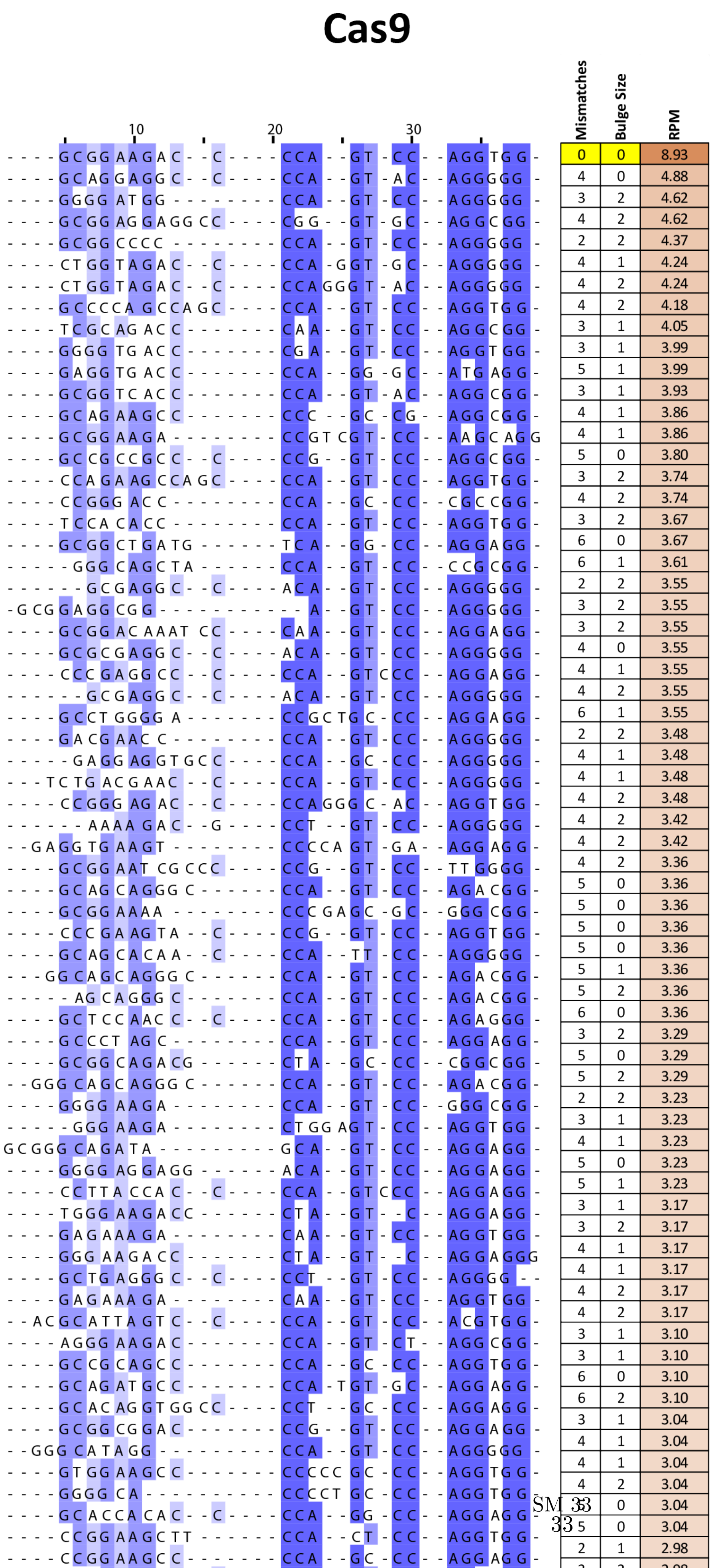

Supplementary Figure 38: Multiple sequence alignment of off-target sites identified by *in vitro* Cas9 CasKAS for the "CD90-1" sgRNA. Shown are the top 100 off-target sites as predicted by Cas-OFFinder and ranked by CasKAS signal. The on-target site (if within the top 100) is highlighted in yellow. The black bars on the bottom indicate the degree of sequence conservation for a given position within the multiple sequence alignment.

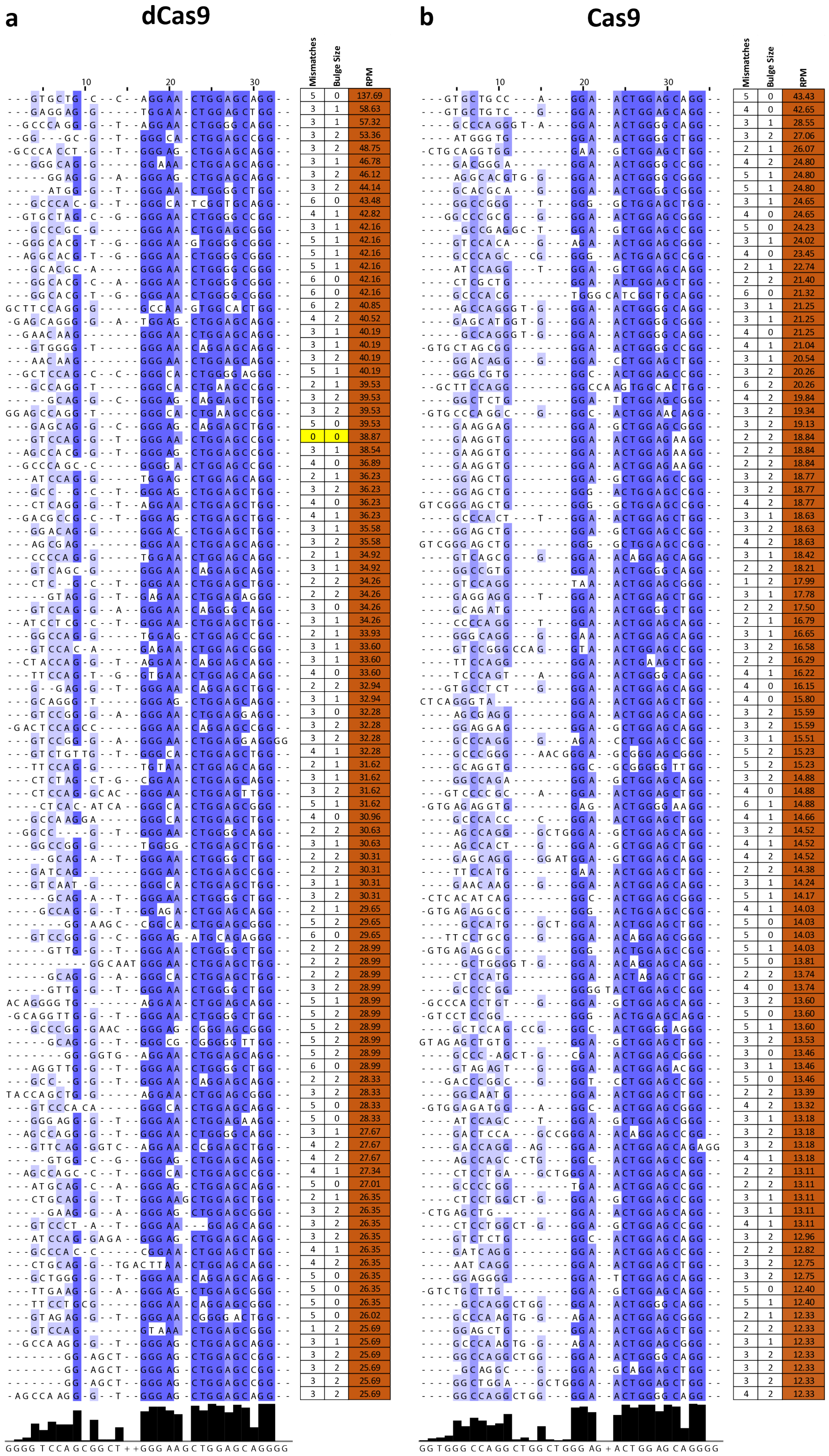

Supplementary Figure 39: Multiple sequence alignment of off-target sites identified by *in vitro* dCas9 and Cas9 CasKAS for the "CD90-2" sgRNA. Shown are the top 100 off-target sites as predicted by Cas-OFFinder and ranked by CasKAS signal. The on-target site (if within the top 100) is highlighted in yellow. The black bars on the bottom indicate the degree of sequence conservation for a given position within the multiple sequence alignment.

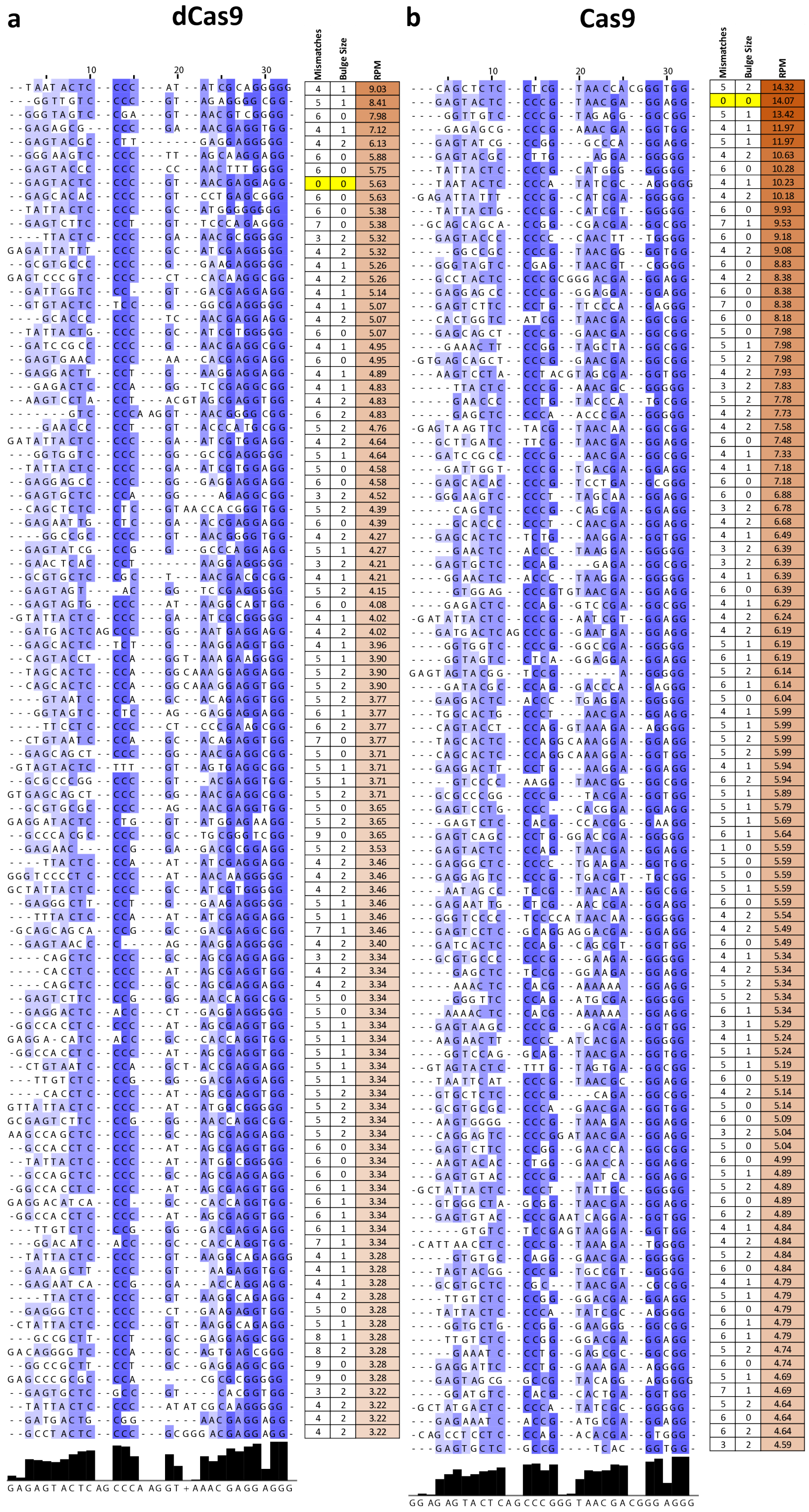

Supplementary Figure 40: Multiple sequence alignment of off-target sites identified by *in vitro* dCas9 and Cas9 CasKAS for the "CD298-1" sgRNA. Shown are the top 100 off-target sites as predicted by Cas-OFFinder and ranked by CasKAS signal. The on-target site (if within the top 100) is highlighted in yellow. The black bars on the bottom indicate the degree of sequence conservation for a given position within the multiple sequence alignment.

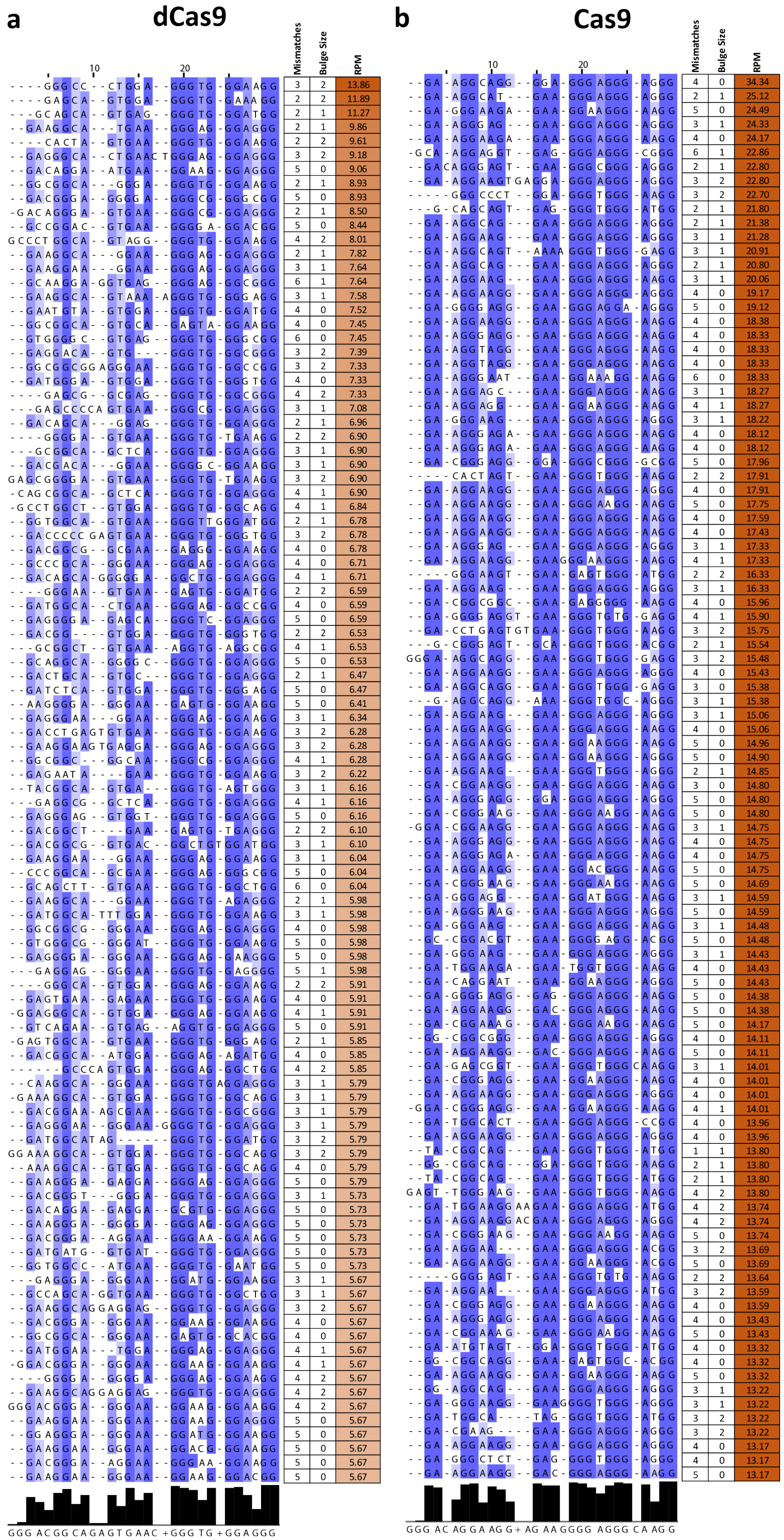

Supplementary Figure 41: Multiple sequence alignment of off-target sites identified by *in vitro* dCas9 and Cas9 CasKAS for the "CD298-2" sgRNA. Shown are the top 100 off-target sites as predicted by Cas-OFFinder and ranked by CasKAS signal. The on-target site (if within the top 100) is highlighted in yellow. The black bars on the bottom indicate the degree of sequence conservation for a given position within the multiple sequence alignment.

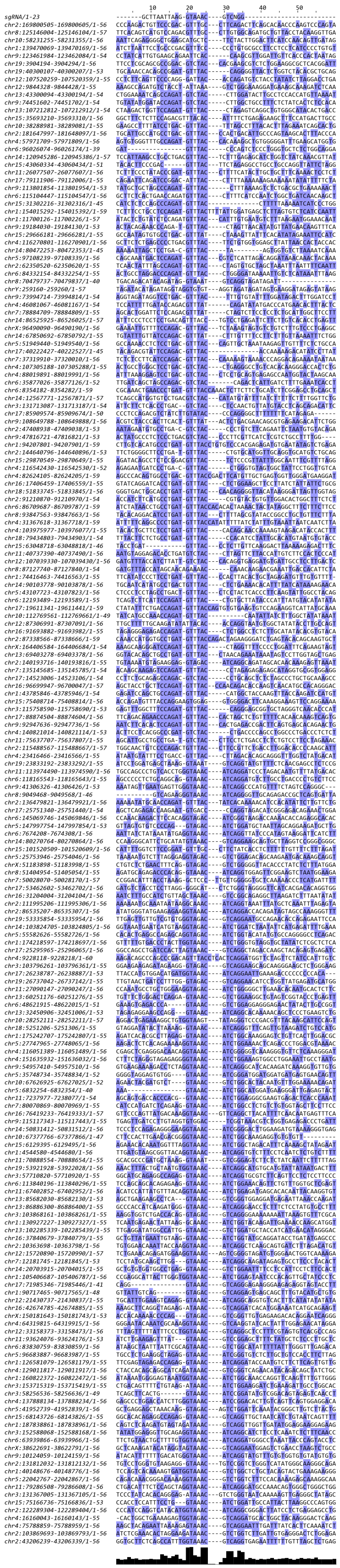

Supplementary Figure 42: Multiple sequence alignment of off-target sites identified by *in vitro* dCas9 and Cas9 CasKAS for the "sgRNA #1" sgRNA outside the list of predicted off-targets by Cass-OFFinder. MACS2 peak calls were manually filtered to exclude artifactual peaks, then the sequence of the ±50-bp region around the peak summit was used as input to the multiple sequence alignment, together with the sgRNA itself.

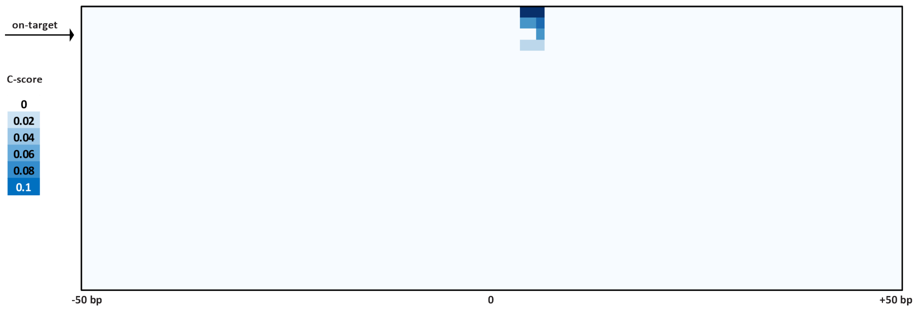

Supplementary Figure 43: Cutting profiles around on- and off-target sites for the VEGFA sgRNA. Four sites where cleavage is observed are identified within the list of predicted off-targets.

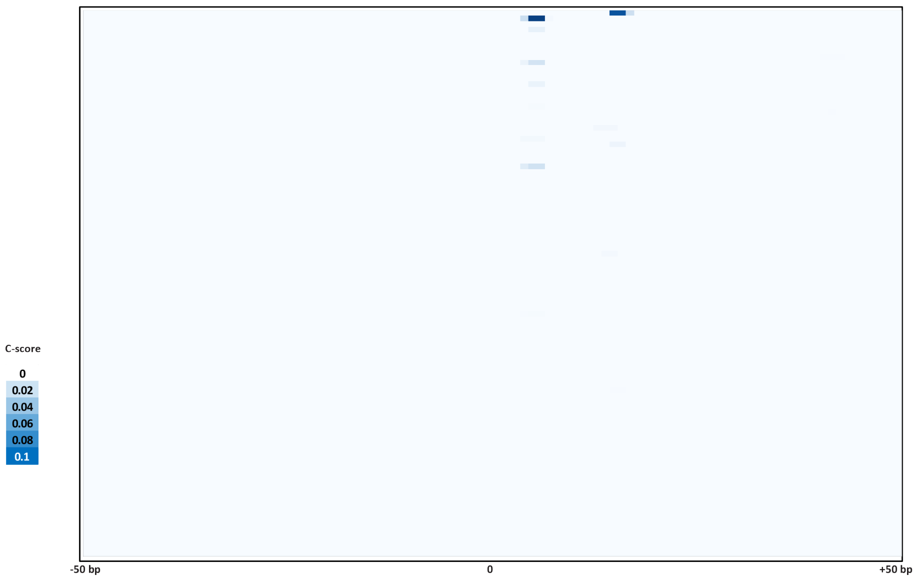

Supplementary Figure 44: Cutting profiles around the top 100 on- and off-target sites for the "CD2-1" sgRNA.

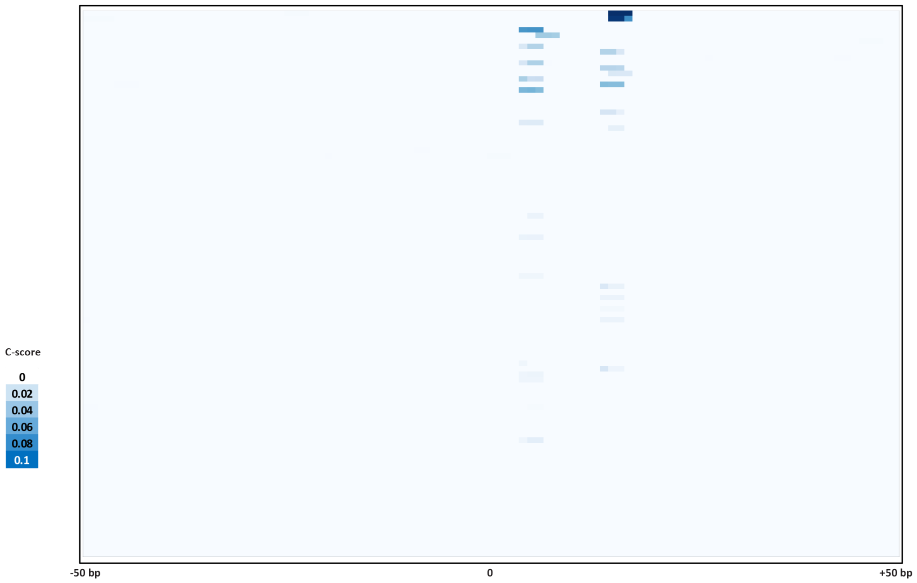

Supplementary Figure 45: Cutting profiles around the top 100 on- and off-target sites for the "CD2-2" sgRNA.

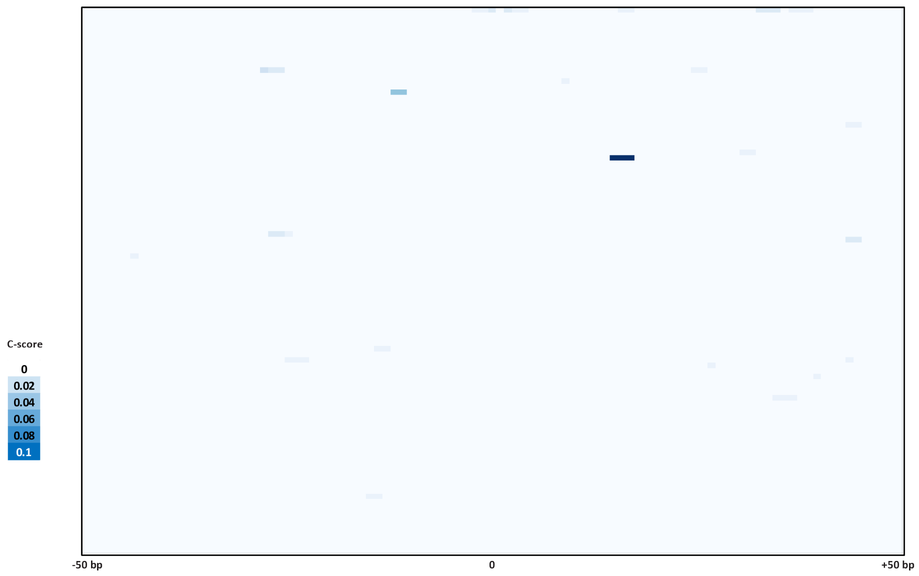

Supplementary Figure 46: Cutting profiles around the top 100 on- and off-target sites for the "CD45-1" sgRNA.

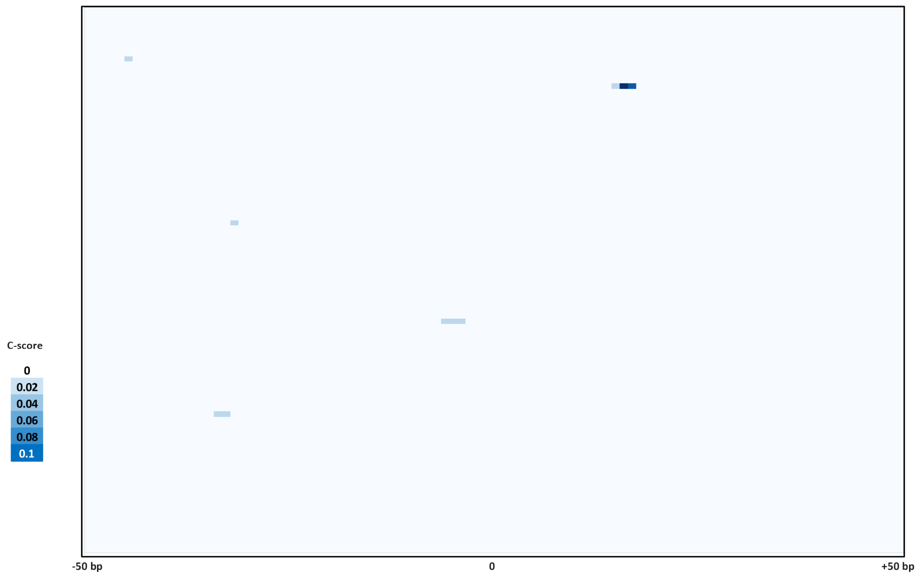

Supplementary Figure 47: Cutting profiles around the top 100 on- and off-target sites for the "CD45-2" sgRNA.

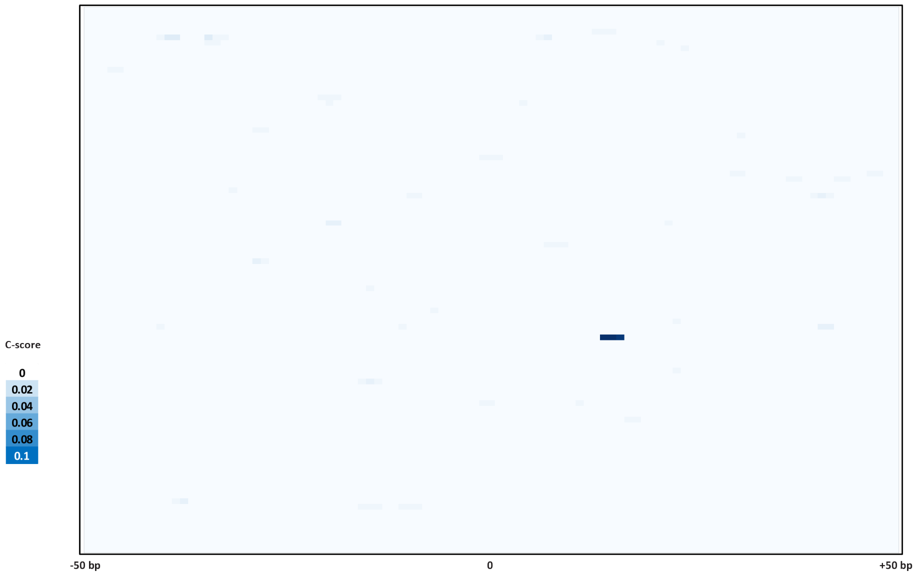

Supplementary Figure 48: Cutting profiles around the top 100 on- and off-target sites for the "CD90-1" sgRNA.

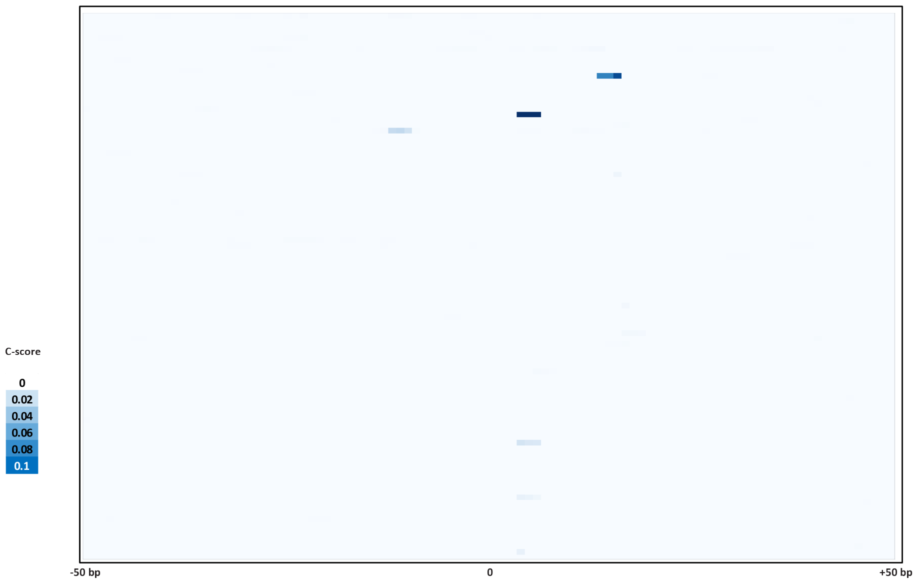

Supplementary Figure 49: Cutting profiles around the top 100 on- and off-target sites for the "CD90-2" sgRNA.

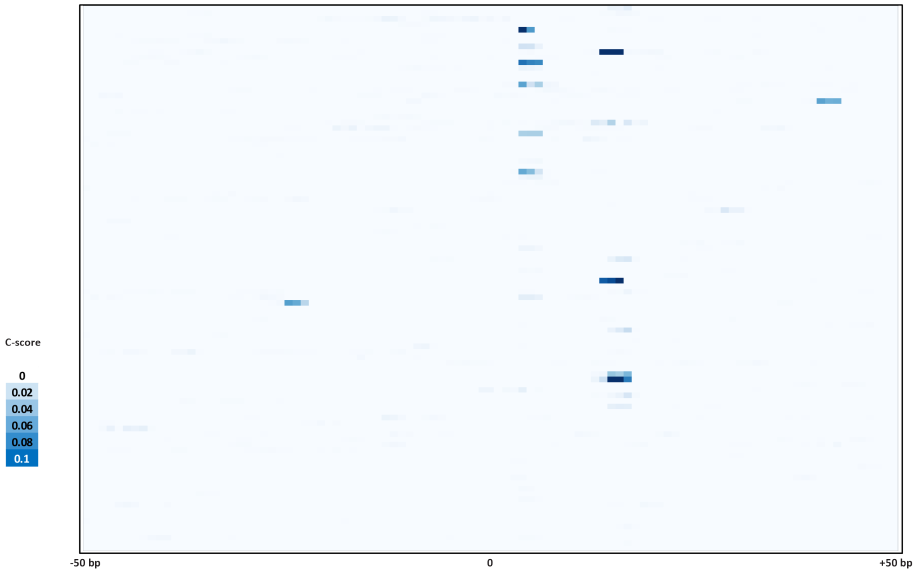

Supplementary Figure 50: Cutting profiles around the top 100 on- and off-target sites for the "CD298-1" sgRNA.

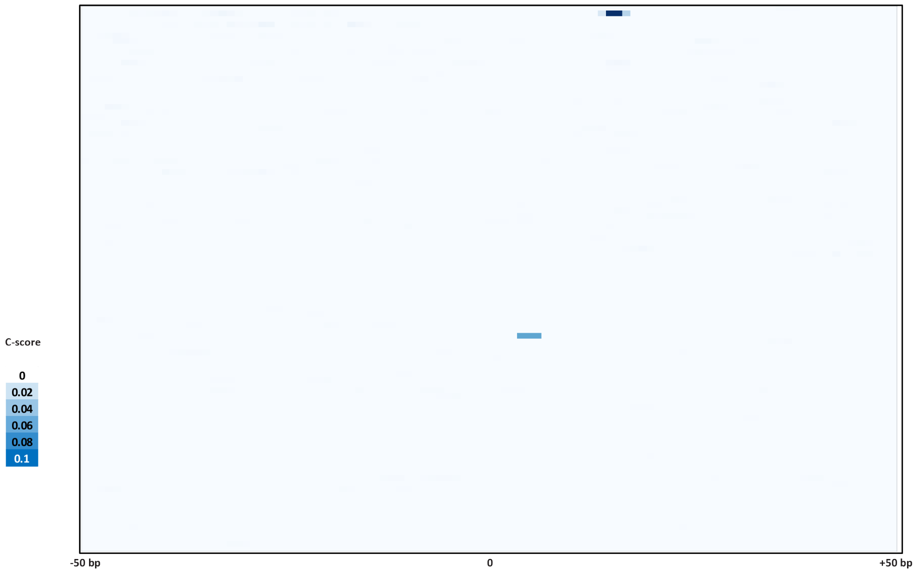

Supplementary Figure 51: Cutting profiles around the top 100 on- and off-target sites for the "CD298-2" sgRNA.

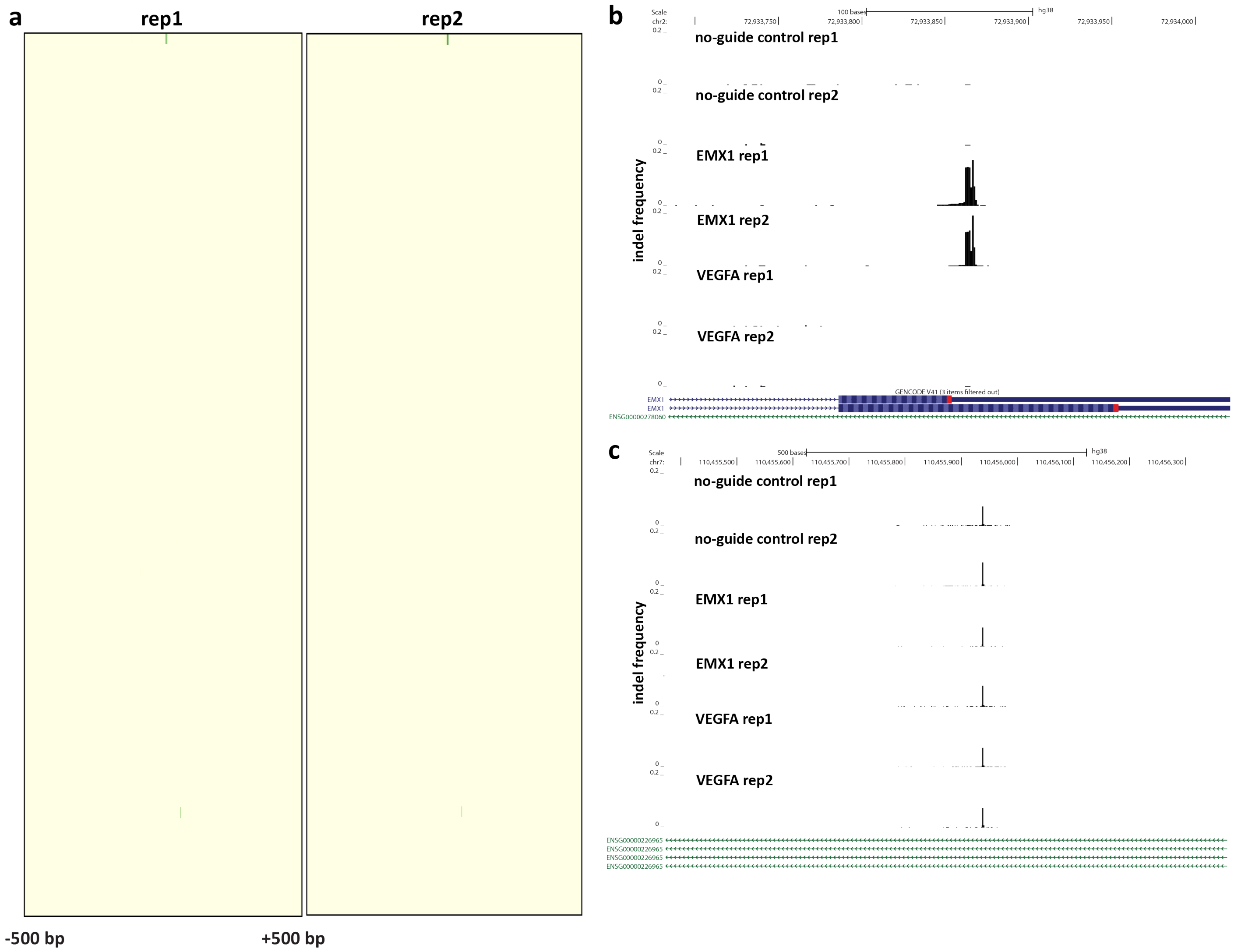

Supplementary Figure 52: Amplicon sequencing of DNA edits with the EMX1 sgRNA. HEK293 cells were transfected (in replicates) with the EMX1 or the VEGFA sgRNAs. Genomic DNA was extracted and a total of 81 potential off-target sites for the EMX1 sgRNA were amplicon-sequenced. (a) The indel frequency (the fraction of reads with an indel over a given position) profiles over each such site identify the on-target site as the only position that is edited, concordant with CasKAS results. (b) Genome browser snapshot of indel frequencies over the on-target site (c) An additional site shows high indel frequency, however, it is present at the same rate in all datasets, including the no-guide negative control, indicating that this is an endogenous sequence variant and not an actual *in vivo* off-target.

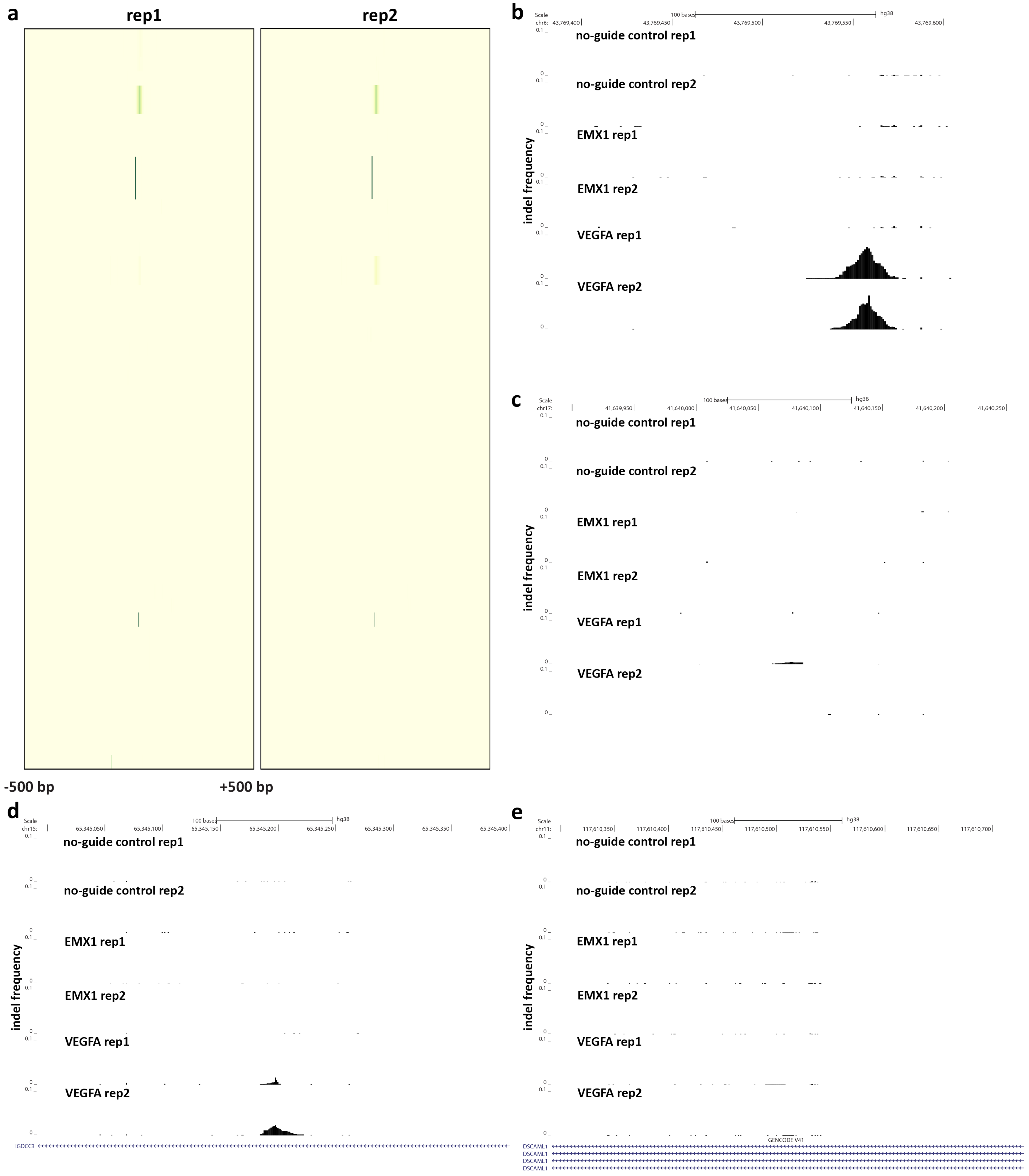

Supplementary Figure 53: Amplicon sequencing of DNA edits with the VEGFA sgRNA. HEK293 cells were transfected (in replicates) with the EMX1 or the VEGFA sgRNAs. Genomic DNA was extracted and a total of 52 potential off-target sites for the VEGFA sgRNA were amplicon-sequenced. Very high *in vivo* indel frequency is observed for the on-target (b) and one of the other sites (d) identified as active Cas9 cutting targets in the *in vitro* CasKAS. Another site (c) also shows elevated indel frequency. The fourth site (e) does not appear to be a cutting target *in vitro*.

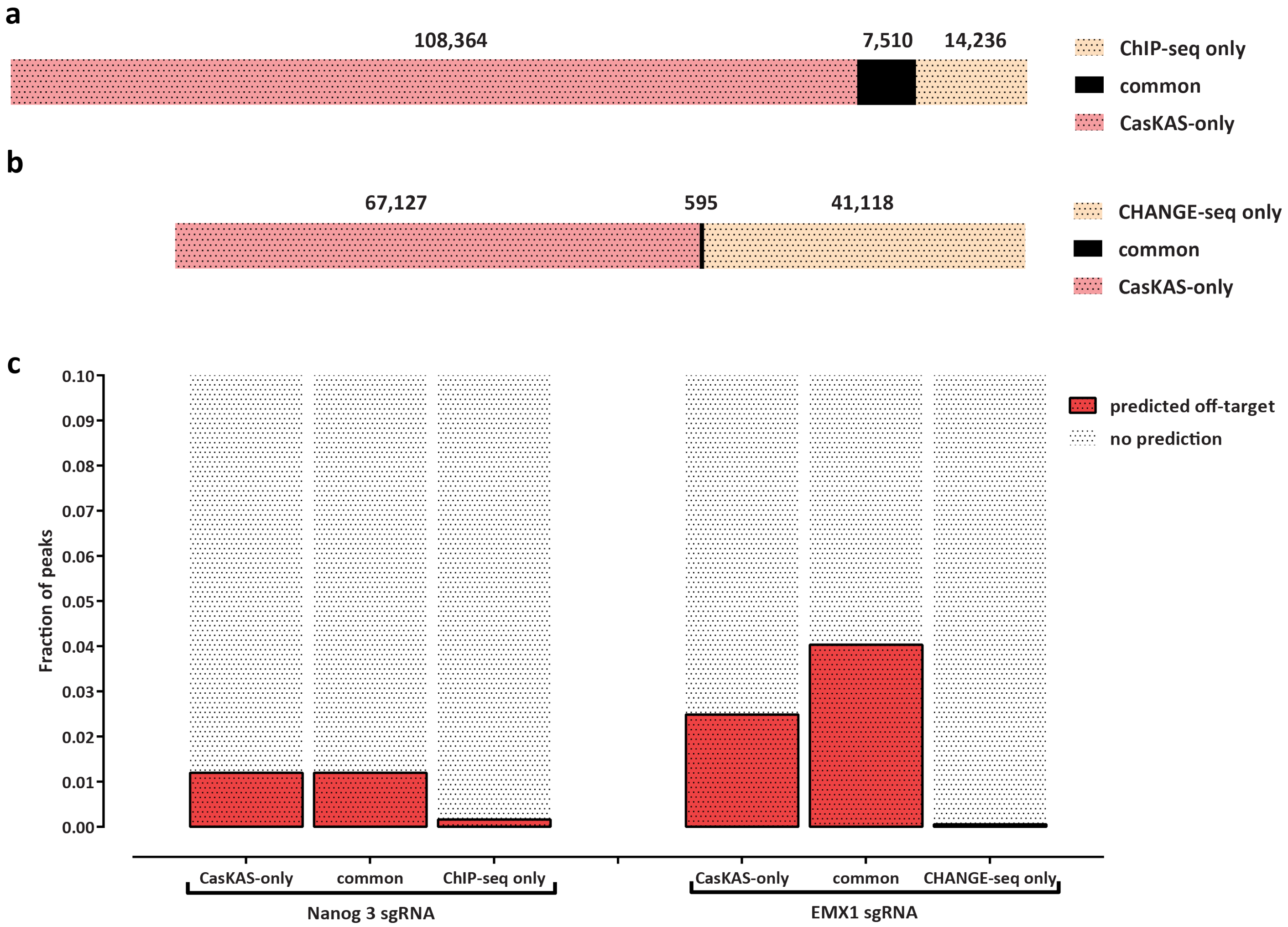

Supplementary Figure 54: Comparing *in vitro* dCas9 results to using ChIP-seq and CHANGE-seq for off-target profiling. Shown is the overlap between MACS2 peak calls for the Nanog-sg3 sgRNA with Nanog ChIP-seq dataset (SRR1168384 from GEO accession ID GSE54745) in (a) and the EMX1 sgRNA with EMX1 CHANGE-seq (SRA accession SRX8227890) in (b). The fraction of peaks common or unique to each assay that are predicted to be off-targets for each sgRNA by Cas-OFFinder is shown in (c).

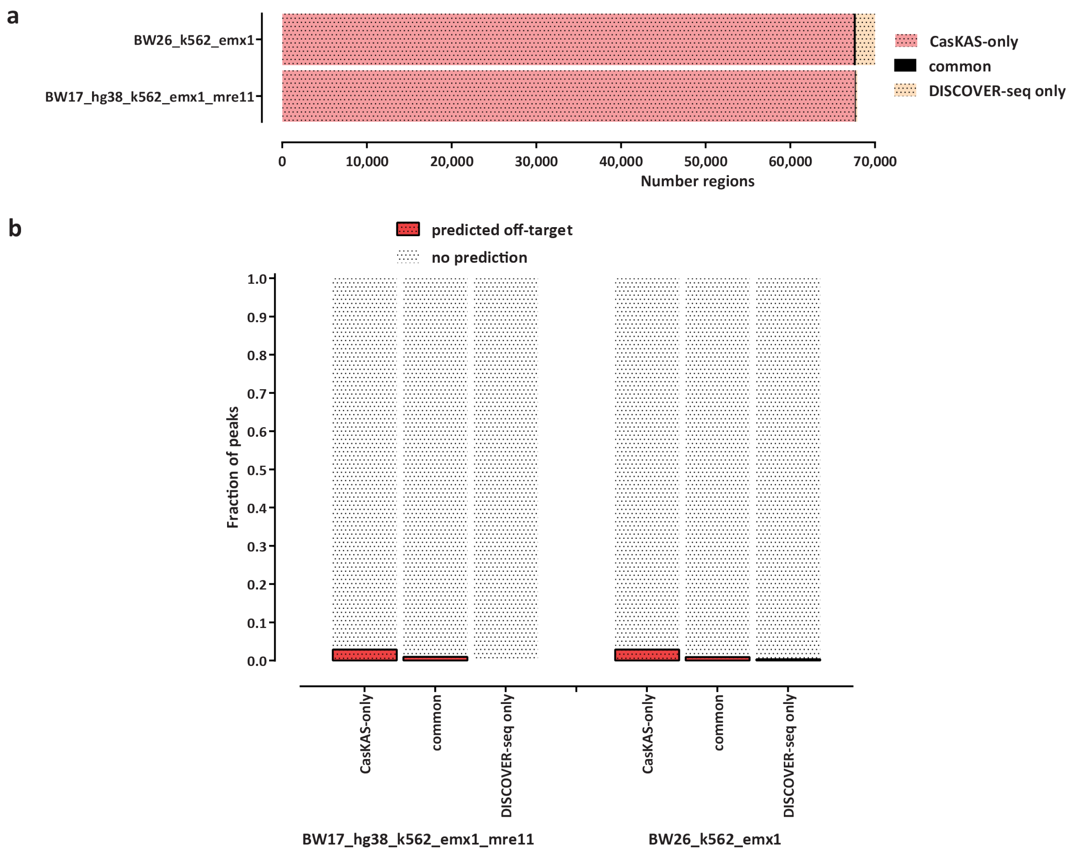

Supplementary Figure 55: Comparing *in vitro* dCas9 results to using DISCOVER-seq for off-target profiling. Shown is the overlap between MACS2 peak calls for the EMX1 sgRNA with MACS2 peak calls on datasets from the original DISCOVER-seq publication[13].

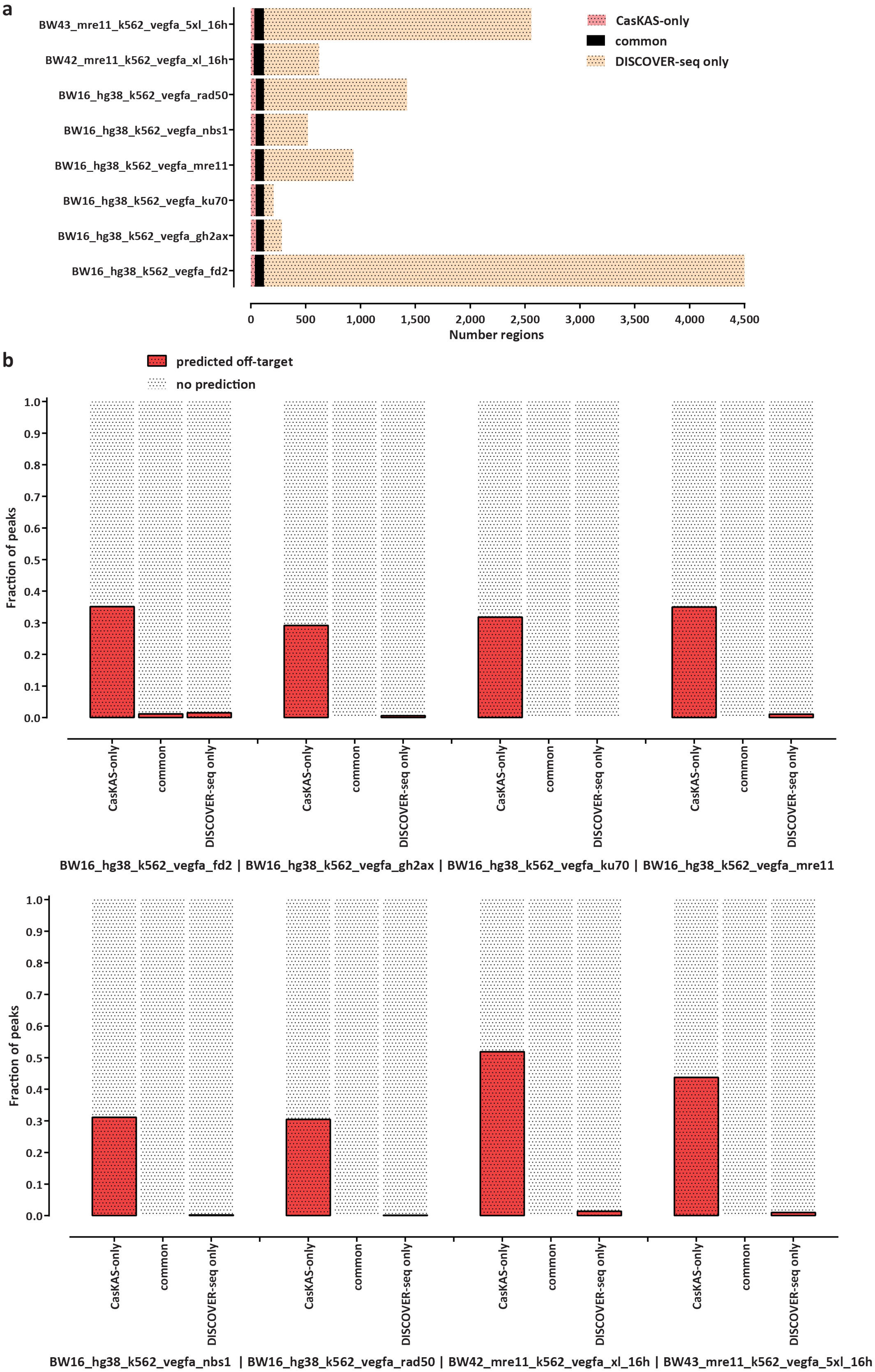

Supplementary Figure 56: Comparing *in vitro* dCas9 results to using DISCOVER-seq for off-target profiling. Shown is the overlap between MACS2 peak calls for the VEGFA sgRNA with MACS2 peak calls on datasets from the original DISCOVER-seq publication[13].

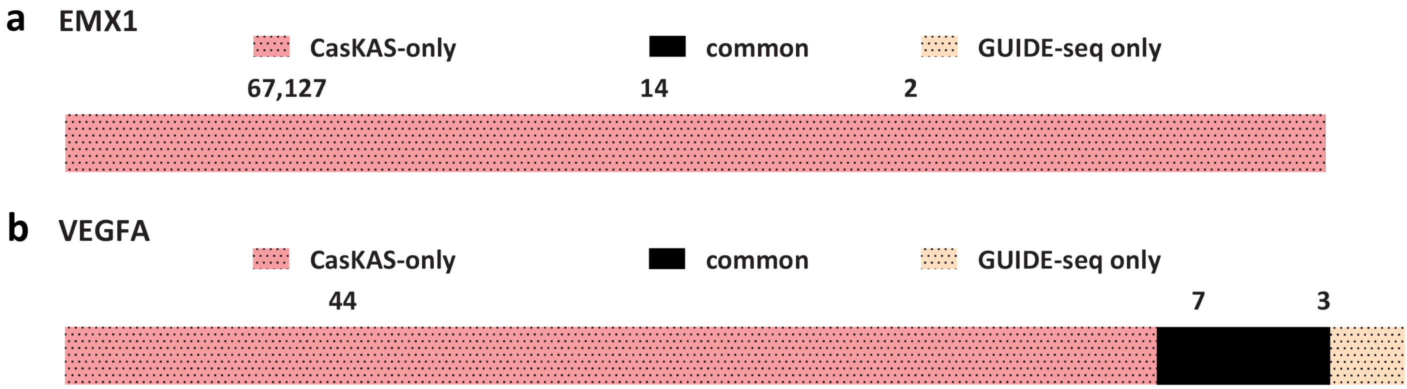

Supplementary Figure 57: Comparing *in vitro* dCas9 results to using GUIDE-seq for off-target profiling. Shown is the overlap between MACS2 peak calls for the EMX1 and VEGFA sgRNAs with off-target regions defined by the original GUIDE-seq publication[4].

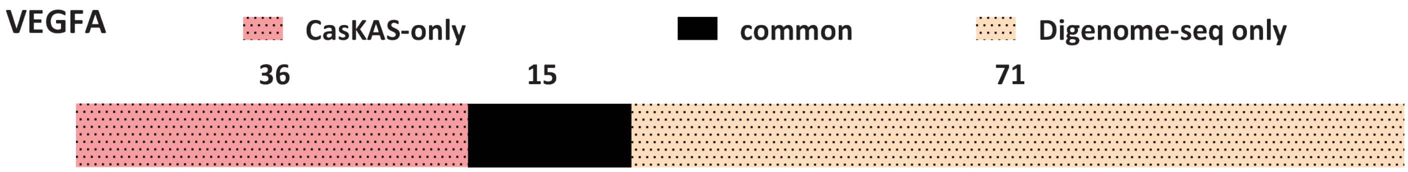

Supplementary Figure 58: Comparing *in vitro* dCas9 results to using Digenome-seq for off-target profiling. Shown is the overlap between MACS2 peak calls for the VEGFA sgRNA with off-target regions defined by the original Digenome-seq publication[2].

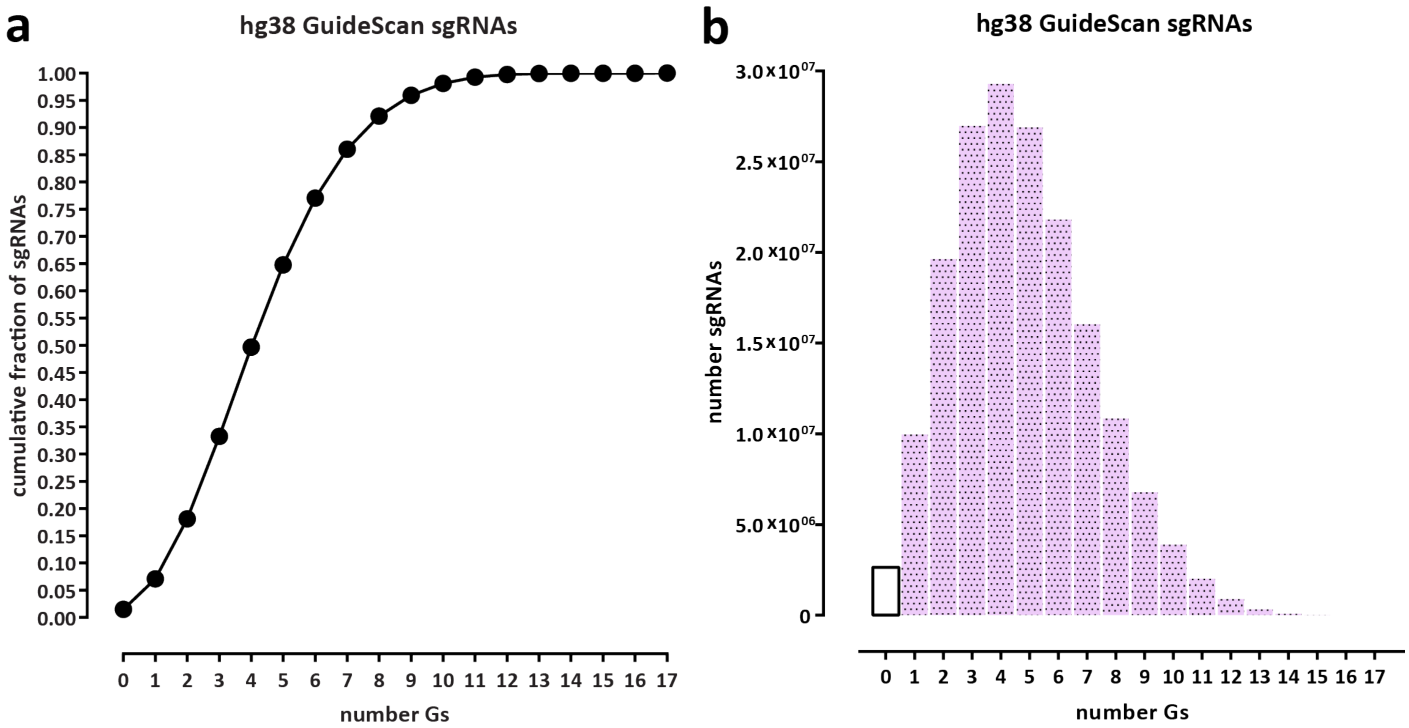

Supplementary Figure 59: Most sgRNAs in the human genome contain multiple G nucleotides and are thus subject to labeling by N_3-kethoxal. Statistics were calculated for all valid sgRNAs as defined by GuideScan[18] (a) Cumulative fraction of sgRNAs. (b) Absolute number of sgRNAs.

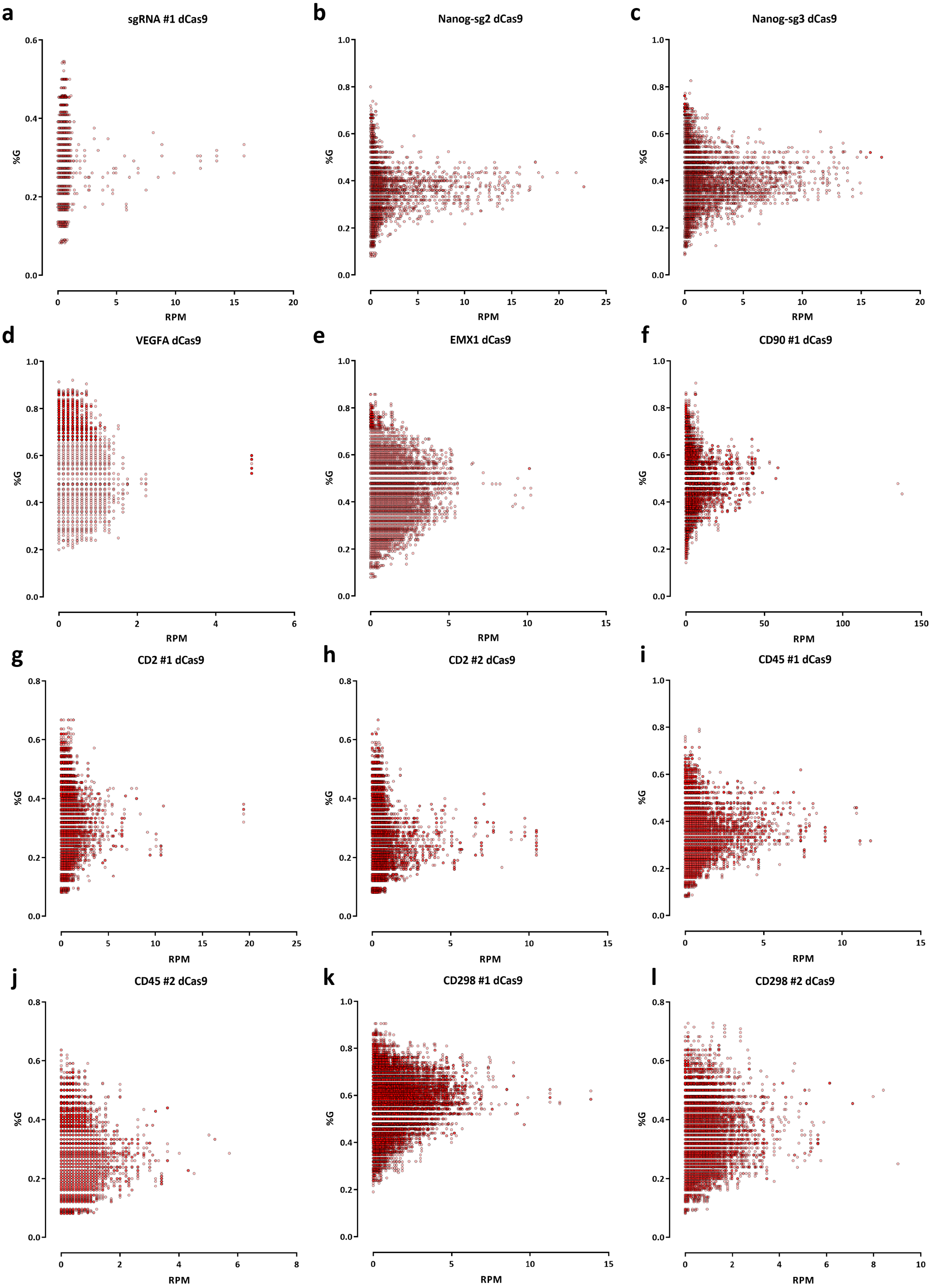
a
sgRNA #1 dCas9
b
Nanog-sg2 dCas9
c
Nanog-sg3 dCas9
d
VEGFA dCas9
e
EMX1 dCas9
f
CD90 #1 dCas9
g
CD2 #1 dCas9
h
CD2 #2 dCas9
i
CD45 #1 dCas9
j
CD45 #2 dCas9
k
CD298 #1 dCas9
l
CD298 #2 dCas9
%G
RPM

Supplementary Figure 60 *(preceding page)*: Absence of strong correlation between the number of G nucleotides in a sgRNA off-target site and CasKAS signal. Highly enriched off-target sites do not show a strong preference for containing more G nucleotides than other predicted off-target sites.
(a) sgRNA #1 dCas9; Pearson r^2 = 0.00, Spearman R = 0.13;
(b) Nanog-sg2 dCas9; Pearson r^2 = 0.10, Spearman R = 0.12;
(c) Nanog-sg3 #1 dCas9; Pearson r^2 = 0.11, Spearman R = 0.18;
(d) VEGFA #1 dCas9; Pearson r^2 = -0.07, Spearman R = -0.09;
(e) EMX1 #1 dCas9; Pearson r^2 = –0.02, Spearman R = –0.06;
(f) CD90 #2 dCas9; Pearson r^2 = 0.07, Spearman R = 0.13;
(g) CD2 #1 dCas9; Pearson r^2 = 0.08, Spearman R = 0.13;
(h) CD2 #2 dCas9; Pearson r^2 = –0.01, Spearman R = –0.01;
(i) CD45 #1 dCas9; Pearson r^2 = 0.07, Spearman R = 0.06;
(j) CD45 #2 dCas9; Pearson r^2 = 0.08, Spearman R = 0.08;
(k) CD298 #1 dCas9; Pearson r^2 = 0.22, Spearman R = 0.28;
(l) CD298 #2 dCas9; Pearson r^2 = 0.06, Spearman R = 0.04.

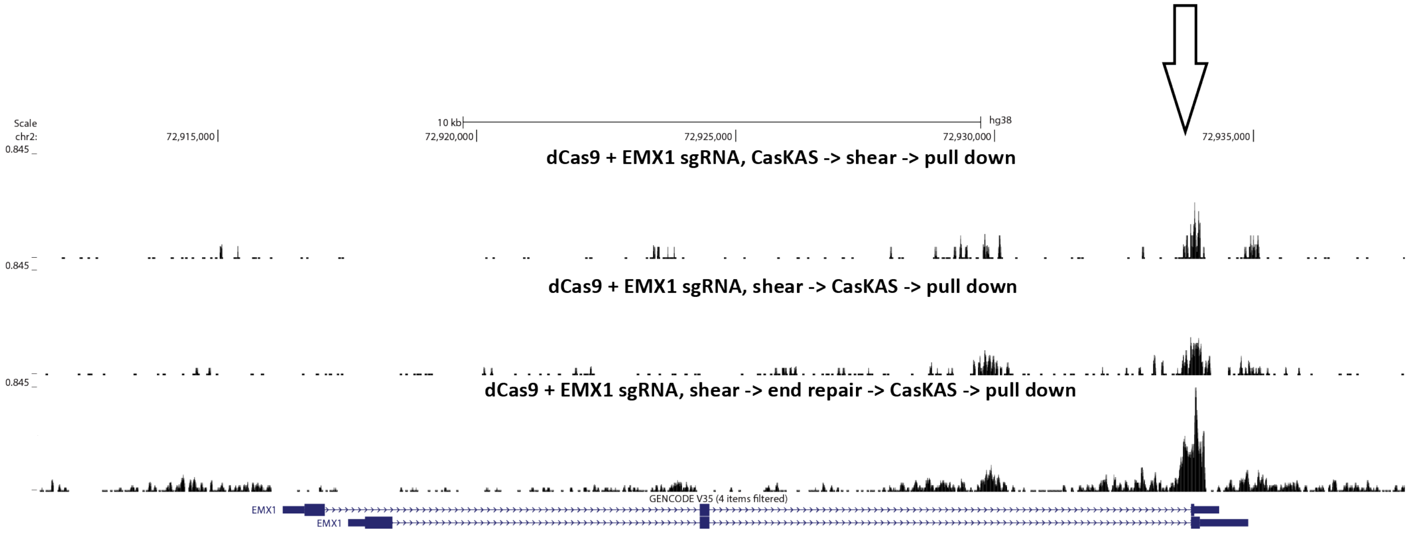

Supplementary Figure 61: CasKAS can be performed on pre-sheared DNA. CasKAS was performed *in vitro* using the EMX1 sgRNA, first, conventionally, by carrying out the CasKAS reaction, then isolating and shearing genomic DNA, and also by pre-shearing the DNA and carrying out the CasKAS reaction on the fragmented DNA. The concern in that case is that the presence of sticky ends containing Gs and unprotected from the action of the N_3-kethoxal would lower the background. This problem can be addressed by carrying out end repair on the sheared DNA prior to the CasKAS reaction.
